# Young plasma-derived exosomes deliver YOD1 to induce M2 polarization and ameliorate osteoporosis via mTOR signaling pathways

**DOI:** 10.7150/ijbs.138620

**Published:** 2026-09-18

**Authors:** Jiahao Wang, Xiaokang Zhang, Siteng Li, Qi Zhang, Jiheng Xiao, Chengbin Huang, Jianpeng Chen, Yang Luo, Zeming Shan, Ziping Chen, Ling Wang, Yingze Zhang

**Affiliations:** 1 Nankai University School of Medicine, Tianjin, China.; 2 Department of Orthopedic Research Center, the Third Hospital of Hebei Medical University, Shijiazhuang, Hebei, P.R. China.; 3 Engineering Research Center of Orthopedic Minimally Invasive Intelligent Equipment, China Ministry of Education.; 4 Key Laboratory of Biomechanics of Hebei Province, Shijiazhuang, Hebei, China.; 5 NHC Key Laboratory of Intelligent Orthopaedic Equipment, Shijiazhuang, Hebei, China.; 6 Key Laboratory of Precise Assessment, Diagnosis, and Treatment of Soft Tissue Injury of Hebei Province; 7 Department of Orthopedic Oncology, The Third Hospital of Hebei Medical University, Shijiazhuang, Hebei, P.R. China.; 8 Department of Orthopedics, Union Hospital, Tongji Medical College, Huazhong University of Science and Technology, Wuhan, Hubei, China.

**Keywords:** osteoporosis, young plasma-derived exosomes, YOD1, macrophage polarization, mTOR signaling pathway

## Abstract

This study aimed to evaluate the therapeutic potential of young plasma-derived exosomes (Y-Exos) in osteoporosis, focusing on their capacity to modulate macrophage polarization and bone homeostasis. Ovariectomized (OVX) mice were administered young plasma, aged plasma, exosome-depleted plasma, or plasma-derived exosomes. Bone mass, macrophage polarization, and osteoblast/osteoclast activity were analyzed by micro-CT, flow cytometry, and histology. Human Y-Exos were isolated to examine their direct effects on BMSC osteogenesis and BMM polarization, as well as indirect effects on osteoclastogenesis. Proteomic and transcriptomic approaches were employed to identify key functional proteins and underlying mechanisms. For results, young plasma significantly improved bone mass, increased CD206^+^M2 macrophages, and upregulated osteogenic and anti-inflammatory genes in OVX mice-effects that were largely abolished upon exosome depletion. Y-Exos were internalized by target cells, promoted BMSC osteogenesis, reduced M1 markers (CD86, iNOS) in LPS-stimulated BMMs, and enhanced M2 markers (CD206, ARG1), and, indirectly, reversed osteoclast activation and rescued osteogenic suppression. *In vivo*, Y-Exos increased bone mass, elevated OCN and CD206 expression, and expanded the Lin^-^Sca-1^+^c-Kit^+^ (LSK) cell population. YOD1 was identified as a key effector in Y-Exos and was downregulated with aging. Mechanistically, Y-Exos delivered YOD1 to activate mTOR in BMMs, driving M2 polarization, and to BMSCs, promoting osteogenesis via Hippo signaling. In conclusion, Y-Exos deliver YOD1 to induce M2 macrophage polarization through mTOR activation and enhance osteogenesis via Hippo signaling, thereby improving the bone immune microenvironment and effectively ameliorating osteoporosis. These findings reveal a new immunomodulatory mechanism and provide an experimental basis for Y-Exos-based therapy in osteoporosis.

## Introduction

Osteoporosis is a prevalent skeletal disorder characterized by reduced bone mineral density (BMD) and impaired bone microarchitecture, resulting in increased bone fragility and susceptibility to fractures. Accumulating evidence indicates that the prevalence of osteoporosis continues to rise worldwide, imposing a substantial burden through increased disability, mortality, and healthcare costs^1^-^4^. Although currently available anti-osteoporotic therapies have shown efficacy in reducing bone loss and fracture risk, their long-term use is constrained by potential adverse effects, such as osteonecrosis of the jaw, atypical femoral fractures, gastrointestinal complications, and cardiovascular events^5^. Moreover, these treatments primarily focus on regulating bone turnover and are insufficient to fully restore the disrupted bone microenvironment. Therefore, the development of novel, effective, and biosafe therapeutic strategies remains an unmet clinical need for osteoporosis management.

Increasing evidence indicates that dysregulation of the osteoimmune microenvironment contributes significantly to the development and progression of osteoporosis^6^, ^7^. The immune system interacts closely with osteoblasts and osteoclasts to regulate bone remodeling and maintain skeletal homeostasis. Under pathological conditions, persistent amplified inflammatory signaling suppresses osteogenic activity and disrupts the balance between osteoblast-mediated bone formation and osteoclast-mediated bone resorption, ultimately shifting bone remodeling toward excessive bone loss^6^, ^7^. Among immune cells, macrophages are key regulators of skeletal homeostasis, as they can adopt distinct functional phenotypes that modulate osteoclast activation and osteogenic repair^8^. The classical classification of macrophages includes pro-inflammatory M1 and anti-inflammatory M2 phenotypes. In estrogen deficiency-induced osteoporosis, the proportion of pro-inflammatory M1 macrophages is increased, whereas that of anti-inflammatory M2 macrophages is reduced, resulting in an imbalanced macrophage polarization profile within the osteoporotic bone microenvironment^9^. M1 macrophages produce abundant reactive oxygen species, nitric oxide, and pro-inflammatory cytokines, including IL-1, IL-6, and TNF-α, which inhibit osteogenic differentiation, promote osteoclast activation, and shift bone remodeling toward excessive bone resorption, thereby disrupting skeletal homeostasis^10^, ^11^. In contrast, M2 macrophages secrete anti-inflammatory cytokines, such as IL-4 and IL-10, inhibit NFATC1-mediated osteoclastogenesis, and facilitate osteogenic differentiation and bone repair^12^. Therefore, targeting macrophage-mediated inflammatory imbalance represents a promising immunomodulatory strategy for restoring bone homeostasis and improving osteoporosis treatment.

Exosomes are extracellular vesicles with a diameter of 30-150 nm^13^. Due to their excellent biocompatibility and ability to deliver functional cargo between cells to regulate recipient cell functions, exosomes have been attractive candidates for regenerative medicine and immunomodulatory therapies^14^. Exosomes derived from bone mesenchymal stem cells (BMSCs) can upregulate the level of the ubiquitin ligase TRIM25, thereby promoting the expression of M2-type markers in bone marrow macrophages^15^. They can also carry microRNA (miR-223) to inhibit the expression of the NLRP3 inflammasome and the apoptotic protein caspase-1 in macrophages, thereby improving macrophage survival under inflammatory conditions^16^. In addition, exosomes derived from umbilical vein endothelial cells, adipose-derived stem cells, and platelet lysates can regulate bone homeostasis through distinct mechanisms and effectively treat osteoporosis^17^-^19^. Recent studies have demonstrated that circulating factors, including plasma-derived exosomes from young individuals, possess regenerative potential^20^-^22^. Parabiosis experiments in animals have shown that blood from young mice can accelerate fracture repair in aged mice^21^. Another study confirmed that exosomes in the plasma of young mice can improve femoral bone mass and alleviate systemic aging manifestations in aged mice^22^. A recent study reported, for the first time, that miR-142-5p in human young plasma exosomes can promote osteogenic differentiation of BMSCs and improve bone mass in osteoporotic rats^23^. However, whether young plasma-derived exosomes can alleviate estrogen deficiency-induced osteoporosis through immunomodulating macrophage polarization remains unclear. Moreover, the functional cargo responsible for their therapeutic effects and the underlying molecular mechanisms have not been fully elucidated.

Therefore, this study aimed to investigate the therapeutic effects of young plasma-derived exosomes in an OVX-induced osteoporosis model and determine whether their effects were mediated through regulation of macrophage polarization and osteoimmune homeostasis. Furthermore, this study sought to identify key functional proteins within young plasma exosomes and elucidate their molecular mechanisms in promoting bone regeneration.

## Methods

### Cells and animals

All mice were purchased from Sipeifu Biotech (Beijing, China) and captured at the animal experiment center of the Third Hospital of Hebei Medical University. All animal experiments obtained the approval of the Medical Ethics Committee of the Third Hospital of Hebei Medical University (Ethics Approval No.: Z2024-039-1).

### Isolation of bone marrow-derived macrophages (BMM)and bone marrow mesenchymal stem cells (BMSC)

After euthanasia, four-week-old female C57BL/6J mice were soaked in 75% alcohol for 5 min for disinfection. The femurs and tibias were dissected and isolated using sterile ophthalmic scissors and forceps, with muscle and connective tissue removed, while maintaining the integrity of the bone tissue to avoid exposing or contaminating the bone marrow. Cut off both ends of the long bones to flush out the bone marrow using a syringe filled with complete medium without M-CSF. After dissociation by pipetting, the suspension was filtered through a 70 μm cell strainer.

**BMM isolation**: The filtered cell suspension was centrifuged at 1000 rpm for 5 min at room temperature. The cells were resuspended in DMEM/F12 Complete Medium (Wuhan Procell Biotechnology Co., Ltd., PM150310B) supplemented with 25 ng/mL M-CSF and seeded in 10 cm culture dishes. After 24 h, non-adherent cells were collected and replated. Half of the medium was replaced with fresh M-CSF-containing medium every 2 days to maintain growth factor levels and remove debris, while leaving adherent cells undisturbed. BMMs were obtained for subsequent experiments once the cell density reached 80% or higher.

**BMSC isolation**: The filtered cell suspension was centrifuged at 1000 rpm for 5 min at room temperature. The cells were resuspended in MEMα Complete Medium (Wuhan Procell Biotechnology Co., Ltd., PM150421B) and seeded in 10 cm culture dishes. After 12 h, non-adherent cells were discarded, and fresh complete medium was added. Subsequently, one-third of the medium was replaced every 2 days until the cell density exceeded 80%. BMSCs were then harvested for subsequent experiments.

### Isolation and characterization of plasma-derived exosomes

Plasma samples from healthy young and aged volunteers were obtained from the Third Hospital of Hebei Medical University, with permission from the Medical Ethics Committee of the Third Hospital of Hebei Medical University (Ethics Approval No.: 2019-021-1). All enrolled volunteers were generally healthy and had no history of chronic diseases or clinically relevant medical conditions. Individuals were excluded if they had acute infections, fever, upper respiratory symptoms, diarrhea, or other inflammatory episodes within the preceding 2 weeks, or if they had autoimmune disorders, malignancies, hematological abnormalities, or long-term immune dysfunction. After obtaining ethical approval and informed consent, whole peripheral blood was collected from healthy volunteers aged 18-35 years, 36-60 years, and 61-75 years (n=6 per group). Y-Exos were collected from the volunteers aged 18-35 years. O-Exos were collected from the volunteers aged 61-75 years. In detail, plasma was obtained by centrifugation at 2000 g for 15 min at 4 °C. Large vesicles, cell debris, and apoptotic bodies were removed from the plasma by gradient centrifugation under the following conditions: 500 g for 5 min at 4 °C; 3000 g for 25 min at 4 °C; and 12000 g for 60 min at 4 °C. The resulting supernatant was collected. Plasma exosomes were extracted using the ExoQuick® Exosome Isolation and RNA Purification Kit (for Serum & Plasma) (EQ806A-1). The supernatant was transferred to a new 1.5 mL EP tube, and the isolation reagent was added at a volume ratio of 500 μL supernatant to 120 μL isolation reagent. After mixing, the mixture was incubated on ice for 30 min, followed by centrifugation at 1500 g for 30 min at 4 °C. The supernatant was discarded, and the yellow precipitate obtained was the exosomes. After resuspension in PBS, quantification was performed using a BCA protein assay kit. The morphology, particle size, zeta potential, and expression of exosomal marker proteins (CD9(+), CD63(+), TSG101(+), Calnexin (-)) were characterized by scanning electron microscopy (SEM), nanoparticle tracking analysis (NTA), and western blot analysis. The uptake of plasma exosomes by BMSCs and BMMs was assessed using an exosome cellular uptake assay. The exosomes were aliquoted and stored at -80 °C.

### Uptake test

BMMs, BMSCs, and RAW264.7 cells (Wuhan Procell Biotechnology Co., Ltd., CL-0190) were seeded at a density of 50,000 cells per dish in glass-bottom cell culture dishes (NEST Biotechnology, 801021). After 24 h of cell culture, exosomes labeled with 100 μg/mL DiI (MedChemExpress, China, HY-D0083) were added to each dish. After 4 h of incubation for cellular uptake, the supernatant was discarded, and uninternalized exosomes were washed away with PBS. Following fixation, nuclei were stained with DAPI working solution (MedChemExpress, China, HY-D0814, blue). After PBS washing, cellular uptake was observed under a confocal microscope (Nikon Instruments, Melville, NY, USA). In addition, BMMs and BMSCs were seeded in 6-well plates at a density of 150,000 cells per well and pretreated with inhibitors targeting different endocytic pathways for 1 h, including 50 μM Dynasore (dynamin activity inhibitor; MedChemExpress, China, HY-15304), 50 μM EIPA (macropinocytosis inhibitor; MedChemExpress, China, HY-101840), and 3 mM MβCD (lipid raft inhibitor; MedChemExpress, China, HY-101461). After pretreatment, exosomes labeled with 100 μg/mL DiD (MedChemExpress, China, HY-D1028) were added to each well. Cells were harvested at 1 h, 2 h, and 4 h after uptake, and the percentage of uptake-positive cells was determined by flow cytometry.

### Cell immune fluorescence staining

Cells were fixed with 4% paraformaldehyde at room temperature for 15 min, then washed three times with PBS. Permeabilization was performed by incubation with 0.3% Triton X-100 (Beijing Solarbio Science & Technology Co., Ltd., T8200) at room temperature for 20 min, followed by blocking with Normal Goat Serum (Beijing Solarbio Science & Technology Co., Ltd., SL038) for 1 h at room temperature. Cells were incubated with appropriately diluted primary antibodies at 4 °C overnight. The primary antibodies were as follows: CD86 Rabbit antibody (Immunoway, USA, YM8023, 1:100), CD206 Rabbit antibody (Immunoway, USA, YM8349, 1:100), iNOS Rabbit antibody (Proteintech, China, 22226-1-AP, 1:100), and ARG1 Rabbit antibody (Abcam, China, ab96183, 1:100). After incubation, cells were washed three times with PBST, then incubated with Goat anti-Rabbit IgG (H+L) Cross-Adsorbed Secondary Antibody, Alexa Fluor™ 594 (Invitrogen, A-11012, 1:2000) and Actin-Tracker Green-488 (Beyotime, C2201S, 1:200) at room temperature for 2 h. After that, cells were washed and incubated with DAPI staining solution (MedChemExpress, China, HY-D0814) at room temperature in the dark for 10 min. After thorough washing with PBS, cells were observed under a fluorescence microscope (IX73, Olympus Corporation, Japan).

### Flow cytometry detection

For bone marrow cells, cells were isolated as previously described for primary mouse bone marrow cell extraction. After centrifugation, cells were collected, and red blood cells were removed using red blood cell lysis buffer (Solarbio, R1010, China). For *in vitro* assays, cells after intervention and thorough washing were collected into flow tubes and directly stained.

For macrophage polarization detection, TruStain FcX™ PLUS (anti-mouse CD16/32, 156604) (Biolegend) was added at 1 μg per 10⁶ cells, followed by incubation on ice for 10 min. Surface marker antibodies were then added and incubated on ice for 1 h. Cells were washed three times with pre-chilled PBS. BD Cytofix™ Fixation Buffer (BD Biosciences, 554655) was added to the cells on ice for 40 min to permeabilize the membranes. Cells were washed three times with 1:10 diluted BD Perm/Wash™ Buffer (10×) (BD Biosciences, 554723), followed by incubation with intracellular antibodies on ice for 1 h. After a final wash with 1× Wash Buffer, samples were analyzed on a CytoFLEX LX flow cytometer (Beckman Coulter, China). The information of antibodies is as follows: PerCP CD86 Antibody (Biolegend, 105025, 1 μg/10^6^cells), PE anti-mouse F4/80(Biolegend, 111603, 1 μg/10^6^ cells), APC CD206 antibody (Biolegend, 321109, 1 μg/10^6^ cells). For detection of Hematopoietic differentiation: APC Sca-1 (ebioscience, 17-5981-83, 1.5 μl/10^6^cells), BV421 CD117 (BD Biosciences, 562609, 1 μl/10^6^cells), PE CD34 (BD Biosciences, 551387, 1 μl/10^6^cells), FITC CD16/32 (BD Biosciences, 561728, 1 μl/10^6^cells), PerCP-Cy5.5 lineage (BD Biosciences, 561317, 20 μl/10^6^cells).

### Immune blotting

Cells or exosome samples were lysed with RIPA buffer containing PMSF (1:100) on ice for 30 min. The supernatant of the lysate was obtained after centrifugation at 10,000 g for 30 min at 4 °C. Protein concentration was determined by BCA assay. The loading buffer was then added at a 1:4 volume ratio, mixed thoroughly, and heated at 100 °C for 5 min. Proteins were separated by SDS-PAGE electrophoresis, followed by membrane transfer, antibody incubation, and chemiluminescent detection to assess the relative expression levels of target proteins. The primary and secondary antibodies are listed as follows:

TSG101 Rabbit antibody (Proteintech, China, 28283-1-AP), CD63 Rabbit antibody (Proteintech, China, 25682-1-AP), CD9 Rabbit antibody (Proteintech, China, 20597-1-AP), Calnexin Rabbit antibody (Proteintech, China, 10427-2-AP), CD86 Rabbit antibody(Immunoway, USA, YM8023), CD206 Rabbit antibody (Immunoway, USA, YM8349), iNOS Rabbit antibody(Proteintech, China, 22226-1-AP), ARG1 Rabbit antibody (Abcam, China, ab96183), RUNX2 Rabbit antibody (Proteintech, China, 20700-1-AP), Osteocalcin Rabbit antibody(Servicebio, China, GB11233-100), ACTIN Mouse antibody(Proteintech, China, 66009-1-Ig), YOD1 Rabbit antibody (Proteintech, China, 25370-1-AP), IDE Rabbit antibody (Proteintech, China, 21728-1-AP), VCL Rabbit antibody (Proteintech, China, 26520-1-AP), GAPDH Rabbit antibody (BOSTER, China, A00227-1), ITCH Rabbit antibody (Proteintech, China, 20920-1-AP), LATS1 Rabbit antibody (Proteintech, China, 17049-1-AP), Phospho-YAP1 (Ser127) Rabbit antibody (Proteintech, China, 80694-2-RR), YAP1 Rabbit antibody (Proteintech, China, 13584-1-AP), Phospho-AKT Rabbit antibody (Proteintech, China, 29163-1-AP), AKT Rabbit antibody (Proteintech, China, 10176-2-AP), Phospho-mTOR Rabbit antibody(ZenBio, China, 381557), mTOR Rabbit antibody (HUA BIO, China, ET1608-5), NFATC1 Mouse antibody (Proteintech, China, 66963-1-Ig).

### QPCR

The total RNA extraction kit (Tiangen Biotech, Beijing, DP419) was used to extract RNA from cells and plasma samples. RNA concentration was quantified using the NanoDrop 2000 spectrophotometer (Thermo Fisher Scientific, USA). cDNA synthesis and qRT-PCR analysis were conducted using the Superbrilliant®6min 1stStrand cDNA Synthesis Kit (Zhongshi TONTRU, China, ZS-M14003) and Superbrilliant® 5×Fast SYBR Green qPCR Mix (Zhongshi TONTRU, China, ZS-M13002). Gene expression levels were calculated using the 2^-ΔΔCt method and normalized to the reference gene GAPDH. Primer sequences were listed in Table 1.

### Osteogenesis, osteoclastogenesis, and macrophage polarisation assays

Osteogenic differentiation induction was performed as follows: Second-passage BMSCs were seeded in culture plates. When cell density reached over 80%, the complete medium was replaced with osteogenic induction medium (For the indirect regulation experiments, the induction medium was replaced with a 1:1 mixture of conditioned medium and osteogenic differentiation medium (Cyagen, OriCell, MUXMX-90021)). Half of the medium was gently replaced every 2 days. On day 7, cells were harvested for detection using an Alkaline Phosphatase Staining Kit (Servicebio, China, G1087-100T), an Alkaline Phosphatase Assay Kit (Beyotime, P0321S), and qPCR analysis. On day 21, cells were stained using an Alizarin Red S Staining Kit (Beyotime, C0148S). For WB detection, cell samples were collected for protein extraction on day 14. Osteoclast differentiation induction was performed as follows: Primary BMMs were seeded in culture plates. When cell density reached over 80%, the complete medium was replaced with osteoclast induction medium (For the indirect regulation experiments, the induction medium was replaced with a 1:1 mixture of conditioned medium and osteoclast induction medium, supplemented with 40 ng/mL RANKL (R&D Systems, USA, 462-TEC)). The medium was changed daily, and fused mature multinucleated osteoclasts could be observed after 7-10 days of induction and detected by TRAP staining (TRAP Staining Kit, Servicebio, China, G1050-50T) and Actin-Tracker Red-555 staining (Beyotime, C2203S). For collecting conditioned medium, BMMs were first stimulated with LPS and subsequently washed and exposed to PBS, Y-Exos, or the corresponding drugs. After treatment, the culture media were collected and designated as conditioned media.

For the LPS-induced macrophage inflammation model, BMMs were treated with 100ng/mL LPS for 24 hours. Thereafter, the medium was replaced with fresh medium containing exosomes or proteins for another 24 hours. After the intervention, the expression of target genes or proteins was detected by qPCR, flow cytometry, immunofluorescence staining, and Western blot analysis.

For signalling inhibition, 2 μM Verteporfin (MedChemExpress, China, HY-B0146), 100 nM Rapamycin (MedChemExpress, China, HY-10219), or 1 μM MK2206 (MedChemExpress, China, HY-108232) were administered with YOD1 or Y-Exos for 24 hours. For osteogenesis, 2 μM Verteporfin was administered to BMSCs with YOD1 or Y-Exos for 2 weeks. For Y-Exos rescue assay, BMM and BMSC were transfected with 40 nM si-YOD1 or si-NC (negative control) using the QM-RNA Plus Transfection Kit (QMTP1001, for immune cells) and QM-RNA Transfection Kit (QMT1001) according to the manufacturer's protocol.

The proteins identified by proteomic analysis were administered to promote osteogenesis and macrophage polarization. The detailed information was as follows: PLS3(Hubei Ipodix Biotechnology Co.,Ltd. PA1000-9177), IDE(MedChemExpress, China, HY-P70291), VCL(MedChemExpress, China, HY-P70994), HPGD (MedChemExpress, China, HY-P75547A), YOD1(Sino Biological Inc., O572-30H), and ITGB2(MedChemExpress, China, HY-P73863).

### Establishment of the ovariectomized (OVX)-induced osteoporosis murine model

As previously described^24^, thirteen-week-old female C57BL/6J mice were selected. After anaesthesia, skin preparation, and disinfection, a skin incision of approximately 0.5-1 cm in length was made along the dorsal midline of the mice, and blunt dissection was performed to expose the dorsal muscles. Muscle tissue was cut with ophthalmic scissors at 1 cm lateral to the spine, and soft tissue was separated bluntly with forceps. The white adipose tissue surrounding the cauliflower-shaped ovary was gently pulled out. The fallopian tube was ligated with suture thread, and the ovary was excised. The remaining adipose tissue was returned to the abdominal cavity, and the peritoneum and muscular layer were sutured, followed by skin closure. Bilateral ovaries were removed sequentially. Penicillin was intramuscularly injected for 2 consecutive days postoperatively to prevent infection.

### Evaluation of different plasma or exosomes treatments *in vivo*

OVX mice were treated with different plasma or exosomes via tail vein injection. Plasma was administered twice weekly at 100 μL per injection for 4 consecutive weeks^25^, ^26^. According to the different treatments, mice were divided into: sham operation group (Sham), OVX group (OVX), OVX + young plasma infusion group (OVX+Plasma-Y), OVX + aged plasma infusion group (OVX+Plasma-O), OVX + young plasma with exosomes-depleted infusion group (OVX+PY-DExos), and OVX + aged plasma with exosomes-depleted infusion group (OVX+PO-DExos) (n=6 per group). For exosome treatment, 100 μg of exosomes in 100 μL was administered every 3 days for 4 weeks. Based on different treatments, mice were divided into the following groups: sham operation group (Sham), OVX group (OVX), OVX + Y-Exos treatment group (OVX+Y-Exos), and OVX + O-Exos treatment group (OVX+O-Exos). After treatment completion, mice were euthanized, and femoral tissues were harvested. Micro-CT (Bruker, Germany) was performed to examine changes in femoral bone mass. Bone volume fraction (BV/TV), bone surface area-to-volume ratio (BS/BV), trabecular number (Tb.N), trabecular separation (Tb.Sp), trabecular thickness (Tb.Th), and BMD were calculated. In addition, HE staining was performed to observe morphological changes in trabecular bone at the femoral metaphysis. Tartrate-resistant acid phosphatase (TRAP) staining was used to detect osteoclastic activation. Immunofluorescence staining was conducted to detect the expression of the osteogenic marker OCN and the anti-inflammatory macrophage differentiation marker CD206. Bone marrow was collected from mouse femurs, and flow cytometry was used to detect the polarization ratio of bone marrow macrophages and the differentiation status of hematopoietic cells.

Among them, young plasma and aged plasma were obtained from 6-week-old and 18-month-old female C57BL/6J mice, respectively. Briefly, after anesthesia, the diaphragm was exposed, and a puncture was made through the diaphragm into the heart. Blood (1 mL) was drawn from the left ventricle using a 1 mL syringe pre-treated with sodium citrate, with gentle manipulation to avoid hemolysis. After removing the needle, the collected blood was transferred into an anticoagulant tube treated with sodium citrate and immediately centrifuged at 2000 g for 15 min at 4 °C. The upper plasma layer was aspirated and used immediately or stored at -80 °C.

### Histological examination

Briefly, femur tissues were fixed and decalcified, then embedded in paraffin and sectioned. After dewaxing with xylene and rehydration with graded ethanol, sections were stained using an H&E staining kit (Servicebio, China, G1076-500mL) and a TRAP staining kit (Servicebio, China, G1050-50T). For H&E staining, sections were dehydrated through graded ethanol, cleared in xylene, mounted in neutral balsam, air-dried at room temperature, and observed under a light microscope. After TRAP staining, sections were mounted with an aqueous mounting medium and observed immediately. The relative area ratio of trabecular bone and the relative area ratio of TRAP-positive regions in the femoral metaphysis were quantified using ImageJ.

### Immunofluorescence staining of tissue

As previously described, bone tissue sections were dewaxed, rehydrated, and antigen-retrieved, followed by blocking with goat serum and permeabilization with 0.5% Triton X-100. After washing with TBST, sections were incubated with primary antibodies at 4 °C overnight. Following TBST washes, fluorescently labeled secondary antibodies were incubated. The secondary antibody was as follows: Goat anti-Rabbit IgG (H+L) Cross-Adsorbed Secondary Antibody (Invitrogen, A-11008, Alexa Fluor™ 488). After thorough washing with TBST, sections were counterstained and mounted using a DAPI-containing mounting medium (Servicebio, China, G1407-25ML). Images were observed under a fluorescence microscope. Target proteins were labeled with fluorescent secondary antibodies, and cell nuclei were stained blue. The relative area percentage of fluorescence-positive regions was analyzed using ImageJ.

### *In vivo* exosomes distribution and biosafety evaluation assays

OVX mouse models were established as described above. Exosomes were labeled with the cell membrane fluorescent dye DiD, and PBS solution containing an equivalent concentration of DiD was prepared. The stained exosomes were washed and collected using a 30 kDa ultrafiltration tube. OVX mice to be observed were divided into three groups: DiD group (DID), DiD-labeled Y-Exos group (Y-Exos), and DiD-labeled O-Exos group (O-Exos), with 6 mice in each group. Each mouse was injected with 100 μL of the corresponding solution. Fluorescence distribution images of bilateral femurs were collected at 6 h, 12 h, 24 h, 36 h, 48 h, and 72 h post-injection. After imaging at 72 h, major organs, including the heart, liver, spleen, lungs, kidneys, and bilateral lower limbs, were harvested for *ex vivo* fluorescence analysis. To evaluate the *in vivo* biosafety of exosomes, mice were intravenously treated with PBS, Y-Exos, or O-Exos. After treatment, the entire peripheral blood sample was collected, and the levels of biochemical indicators, including urea (UREA), uric acid (UA), albumin (ALB), alanine aminotransferase (ALT), creatinine (CRE), and aspartate aminotransferase (AST), were measured at Ruiweiyouke Company. In addition, major organs, including the heart, liver, spleen, lungs, and kidneys, were harvested, and HE staining was performed to assess whether exosome treatment affected the histological morphology of these organs in mice.

### Proteomic and RNA sequencing analysis

The protein profiles of Y-Exos and O-Exos were characterized by proteomic analysis. Exosomal protein samples were first lysed with RIPA buffer and subsequently subjected to mass spectrometric analysis using the timsTOF platform, with the sequencing service provided by OE Biotech (China). In parallel, RNA-seq was performed to characterize transcriptional changes in cells following LPS exposure in the presence or absence of YOD1. Briefly, cellular RNA was isolated and used for cDNA library preparation, and the resulting libraries were sequenced using the Illumina NovaSeq 6000 system with technical assistance from OE Biotech, Inc. (Shanghai, China).

### Statistical analysis

Quantitative data obtained from the *in vitro* and *in vivo* experiments are expressed as mean ± SD. Each statistical analysis included at least three independent biological replicates. ImageJ software was utilized for densitometric measurement of protein levels and determination of the area of positive staining. All statistical analyses were performed with GraphPad Prism 8 (GraphPad Software, USA). Differences between two independent groups were assessed using an unpaired Student's t-test, while comparisons among multiple groups were conducted by one-way ANOVA with Tukey's post hoc test. A two-sided P value below 0.05 was considered statistically significant, whereas NS denotes a non-significant difference.

## Results

This study investigated the effects of young and aged plasma treatments on bone marrow macrophage differentiation and bone mass regulation in ovariectomized (OVX) mice. Thirteen-week-old female C57BL/6 mice were subjected to OVX to establish an osteoporosis model and then treated with young plasma (Plasma-Y), aged plasma (Plasma-O), exosome-depleted young plasma (PY-DExos), and exosome-depleted aged plasma (PO-DExos), respectively. The experimental procedure is illustrated in Figure 1(A). Flow cytometry was performed to determine the proportions of M1 (F4/80^+^CD86^+^) and M2 (F4/80^+^CD206^+^) macrophages in bone marrow. As shown in Figure 1(B-C), compared with the sham group, the percentage of CD206^+^ macrophages in the OVX group was significantly decreased from 15.0% to 4.8%, whereas the increase in CD86^+^ macrophages from 6.0% to 7.6% was not statistically significant. Compared with the OVX group, Plasma-Y treatment markedly increased the proportion of CD206⁺ macrophages from 4.8% to 13.0% without significantly affecting CD86^+^ macrophages. Plasma-O treatment also mildly elevated the percentage of CD206^+^ macrophages from 4.8% to 6.5% but had no significant influence on CD86^+^ macrophages. After exosome depletion, the ability of both PY-DExos and PO-DExos to increase CD206^+^ macrophages was significantly attenuated, while neither treatment significantly affected CD86^+^ macrophages. Consistently, Plasma-Y significantly increased the CD206^+^/CD86^+^ ratio, and this effect was notably weakened after exosome removal. Plasma-O exerted no significant effects.

Furthermore, qPCR detected the mRNA expression levels of genes associated with osteogenic differentiation and macrophage polarization in peripheral blood. As shown in Figure 1(D), osteogenic markers OCN and RUNX2 showed significantly lower mRNA expression in the OVX group compared with the sham group, whereas the expression of COL-I remained unchanged. Compared with the OVX group, Plasma-Y treatment significantly upregulated the expression levels of COL-I and OCN, and these effects were weakened after exosome depletion, with only COL-I remaining significantly increased. In contrast, neither Plasma-O nor PO-DExos significantly affected the expression of the osteogenic genes examined. With respect to macrophage polarization-related genes (Figure 1(E)), compared with the sham group, IFN-γ expression was significantly upregulated, whereas IL-4 and ARG1 expression was significantly downregulated in the peripheral blood of OVX mice. Compared with the OVX group, Plasma-Y treatment significantly reduced IFN-γ expression and increased IL-4 and Arg1 expression. PY-DExos treatment also decreased IFN-γ and elevated IL-4 levels, although these effects were weaker than those observed with Plasma-Y. Notably, Plasma-O significantly upregulated IFN-γ expression, whereas PO-DExos had no significant effect on the macrophage polarization-related genes.

Micro-CT imaging was performed to evaluate bone mass changes in the proximal femur. Coronal sections and three-dimensional reconstructed images of the femoral metaphysis are presented in Figure 2(A). Compared with the sham group, the proximal femur of OVX mice exhibited typical osteoporotic morphological features, including a markedly sparse and fractured trabecular bone network, reduced trabecular connectivity, and disrupted structural integrity. Plasma-Y significantly improved trabecular continuity and density and increased metaphyseal bone mass, whereas Plasma-O further aggravated trabecular sparsity and reduced bone mass. After exosome depletion, PY-DExos and PO-DExos retained the positive and negative effects of Plasma-Y and Plasma-O, respectively, although these effects were attenuated. Statistical analyses of bone mass and bone structural parameters are shown in Figure 2(B). Compared with the sham group, OVX significantly decreased BV/TV, BMD, Tb. N, and Tb. Th and increased BS/BV and Tb.Sp. Compared with the OVX group, Plasma-Y significantly increased BV/TV, BMD, and Tb. N and reduced BS/BV and Tb. Sp, without significantly affecting Tb.Th. In contrast, Plasma-O significantly decreased BMD and increased BS/BV and Tb. Sp, with no significant effects on the other parameters. After exosome depletion, the effects of Plasma-Y on BV/TV and BMD were significantly attenuated, whereas the effects of Plasma-O remained largely unchanged. PY-DExos still significantly improved BV/TV and BMD and reduced Tb. Sp, while PO-DExos significantly increased Tb.Sp without significantly affecting the other parameters.

Further histological changes and osteoclast activity in the proximal femur were examined by HE staining and TRAP staining (Figure 2C-E, H(a-b)). Compared with the Sham group, the OVX group exhibited sparse trabecular bone, increased vacuolar adipocyte infiltration, reduced normal hematopoietic tissue, and increased TRAP-positive areas around the subchondral bone and trabeculae, indicating enhanced osteoclast activity. Compared with the OVX group, Plasma-Y significantly increased trabecular number, reduced bone marrow adipocyte infiltration, and suppressed osteoclast activation. In contrast, Plasma-O further decreased trabecular number, aggravated bone marrow fatty infiltration, and enhanced osteoclast activity in the subchondral bone region, but not in the trabecular region. After exosome depletion, PY-DExos showed a slight increase in trabecular number and reduced osteoclast activation, although these changes were not statistically significant, whereas PO-Dexos had no significant effects on either parameter.

Immunofluorescence staining was performed to detect OCN expression in the proximal femur and CD206 expression in the bone marrow (Figure 2F-G, H(c-e)). Compared with the sham group, the OVX group exhibited significantly decreased OCN fluorescence intensity around the subchondral bone and trabeculae, together with reduced CD206 expression in the bone marrow. Plasma-Y significantly enhanced OCN and CD206 expression, while PY-DExos retained a significant promoting effect on CD206 but not on OCN expression. In contrast, neither Plasma-O nor PO-DExos significantly affected OCN or CD206 expression.

To determine whether plasma-derived exosomes regulate osteogenesis and macrophage polarization, Y-Exos and O-Exos were isolated from fresh peripheral plasma of young and old volunteers, respectively. As shown in Figure 3(A), SEM analysis revealed that both Y-Exos and O-Exos exhibited typical vesicular morphology. Uptake assays confirmed that both exosome preparations could be internalized by primary mouse BMSCs, BMMs, and RAW264.7 cells. NTA analysis showed comparable particle sizes between Y-Exos and O-Exos, with average diameters of 100.4 nm and 101.6 nm, respectively.

Western blotting further confirmed the expression of exosomal markers TSG101, CD63, and CD9 in both preparations. The effects of Y-Exos and O-Exos on osteogenic differentiation of BMSCs were subsequently evaluated. As shown in Figure 3(B-C, F(a-b)), Y-Exos significantly promoted ALP activity and mineralized nodule formation, whereas O-Exos showed no significant effect. Consistently, qPCR analysis demonstrated that Y-Exos markedly upregulated the expression of osteogenic genes COL-I, RUNX2, and OCN in BMSCs, while O-Exos did not significantly alter their expression (Figure 3G). The effects of Y-Exos and O-Exos on osteoclast differentiation were assessed using BMMs. Y-Exos did not significantly affect osteoclast formation, whereas O-Exos significantly increased osteoclast area and the average number of nuclei per osteoclast, as shown by TRAP and F-actin ring staining (Figure 3D-F(c-d)). Furthermore, the effects of exosomes on macrophage polarization were examined under non-inflammatory and LPS-induced inflammatory conditions (Figure 3H-J). Under non-LPS conditions, neither Y-Exos nor O-Exos significantly affected the expression of M1 markers CD86 and iNOS. However, Y-Exos significantly increased the expression of M2 markers CD206 and ARG1, whereas O-Exos showed no significant effect. Under LPS stimulation, Y-Exos reduced the expression of M1 markers and increased M2 marker expression. In contrast, O-Exos did not significantly alter macrophage polarization under inflammatory conditions.

To further investigate whether plasma-derived exosomes regulate bone remodeling through macrophage polarization, flow cytometry was performed to evaluate changes in BMM polarization. Under non-LPS conditions, neither Y-Exos nor O-Exos significantly affected the proportion of CD86⁺ BMMs. However, Y-Exos significantly increased the proportion of CD206⁺ BMMs from 38.8% to 44.7%, whereas O-Exos showed no significant effect (Figure 4A-B). Under LPS-induced inflammatory conditions, LPS increased M1 polarization and decreased M2 polarization. Y-Exos significantly reduced the proportion of M1-polarized BMMs and increased M2 polarization, whereas O-Exos did not significantly alter macrophage polarization. To further evaluate the indirect effects of exosome-regulated macrophages on bone cells, conditioned medium from treated BMMs was collected and used to induce osteoclastic differentiation of BMMs and osteogenic differentiation of BMSCs, as illustrated in Figure 4(C). TRAP and F-actin ring staining (Figure 4D, F(a-b)) revealed that conditioned medium from LPS-stimulated BMMs (CM+LPS) significantly promoted osteoclast differentiation and maturation, as indicated by increased osteoclast area and nuclei number (Figure 4D, F(a-b)). Conditioned medium from Y-Exos-treated BMMs (CM+LPS+Y-Exos) markedly suppressed this pro-osteoclastic effect, whereas CM+LPS+O-Exos showed no significant improvement. Furthermore, ALP and ARS staining (Figure 4E, F(c-d)) demonstrated that CM+LPS significantly inhibited osteogenic differentiation of BMSCs compared with control conditioned medium (CM) (Figure 4E, F(c-d)). CM+LPS+Y-Exos partially reversed the inhibitory effect of CM+LPS on osteogenic differentiation, whereas CM+LPS+O-Exos failed to restore osteogenic differentiation. Consistently, qPCR analysis revealed that CM+LPS suppressed the expression of osteogenic genes COL-I, RUNX2, and OCN, while Y-Exos significantly alleviated this suppression. In contrast, O-Exos showed no significant effect on LPS-induced inhibition of osteogenic gene expression (Figure 4G).

To investigate the mechanism underlying plasma-derived exosome uptake by BMMs and BMSCs, Y-Exos were labeled with DiD and incubated with cells in the presence of different endocytosis inhibitors, including Dynasore (a dynamin inhibitor), EIPA (a macropinocytosis inhibitor), and MβCD (a lipid raft inhibitor). As shown in Figure 5(A-B), the proportion of DiD-positive BMMs and BMSCs gradually increased over time after Y-Exos incubation, indicating efficient cellular internalization. Compared with BMSCs, BMMs exhibited a higher uptake capacity for Y-Exos. Dynasore treatment significantly reduced Y-Exos uptake in both BMMs and BMSCs at different time points, with a stronger inhibitory effect observed in BMMs, suggesting that dynamin-dependent endocytosis is a major pathway involved in Y-Exos internalization. In contrast, EIPA and MβCD showed relatively weaker and variable effects on Y-Exos uptake, indicating that macropinocytosis and lipid raft-mediated pathways may contribute to exosome internalization but are not the predominant mechanisms.

To further determine whether exosome uptake is required for the biological effects of Y-Exos, Dynasore was used to inhibit exosome internalization, followed by evaluation of macrophage polarization and osteogenic differentiation-related proteins (Figure 5C--D). Dynasore pretreatment partially reversed the inhibitory effect of Y-Exos on the proinflammatory marker iNOS and reduced the promoting effect of Y-Exos on the anti-inflammatory marker ARG1 in BMMs under inflammatory conditions. It also partially abrogated the upregulatory effect of Y-Exos on the osteogenic differentiation proteins RUNX2 and OCN in BMSCs. Notably, Dynasore alone did not exert an obvious effect on BMM polarisation or BMSC osteogenic differentiation. These results indicate that Y-Exos uptake through dynamin-dependent endocytosis is required, at least partially, for their regulatory effects on macrophage polarization and osteogenic differentiation.

To investigate the *in vivo* distribution of plasma-derived exosomes, DiD-labeled Y-Exos and O-Exos were intravenously injected into OVX mice, and fluorescence signals were monitored using an *in vivo* imaging system (IVIS). As shown in Figure 6(A-B), fluorescence signals were detected in the bilateral femoral regions as early as 6 h after injection. Compared with free DiD, both Y-Exos and O-Exos exhibited stronger and more sustained accumulation in the femur, indicating enhanced bone distribution and retention of exosomes. Femoral fluorescence intensity increased over time and reached a peak around 12 h after injection. Although Y-Exos showed a higher fluorescence intensity than O-Exos at several time points, the difference was only significant at 36 h, suggesting a relatively enhanced retention capacity of Y-Exos in the femoral region. *Ex vivo* fluorescence imaging at 72 h after injection further demonstrated that the liver showed the strongest fluorescence signals among the examined organs in all groups (Figure 6C-D). Compared with free DiD, both Y-Exos and O-Exos exhibited increased accumulation in the liver, whereas no significant differences were observed in the distribution of other major organs. Importantly, fluorescence intensity in isolated femurs remained significantly higher in the Y-Exos and O-Exos groups than in the free DiD group, confirming the sustained accumulation of plasma-derived exosomes in bone tissue. To evaluate the potential systemic toxicity of plasma-derived exosomes, histological examination of major organs and serum biochemical analyses were performed (Supplementary Figure S1). Neither Y-Exos nor O-Exos induced obvious pathological changes in major organs or altered hepatic and renal function-related parameters, including urea (UREA), uric acid (UA), albumin (ALB), alanine aminotransferase (ALT), creatinine (CRE), and aspartate aminotransferase (AST). These results demonstrate that plasma-derived exosomes can efficiently accumulate in the femur of OVX mice and exhibit favorable *in vivo* biocompatibility without detectable systemic toxicity.

We further investigated the therapeutic effects of plasma-derived exosomes on osteoporosis in OVX mice. After 4 weeks of treatment, micro-CT analysis revealed that Y-Exos significantly improved trabecular continuity and density and increased femoral metaphyseal bone mass, whereas O-Exos did not produce significant changes (Figure 7A). Quantitative analysis of bone parameters showed that Y-Exos significantly increased BV/TV, BMD, Tb. N, and Tb. Th, while reducing BS/BV and Tb.Sp. In contrast, O-Exos showed no significant effects on these parameters (Figure 7B). Histological analysis further confirmed the protective effects of Y-Exos on bone structure. HE staining showed that Y-Exos increased trabecular bone formation and reduced bone marrow adipocyte infiltration, whereas O-Exos did not significantly affect trabecular morphology (Figure 7C, G(a)). TRAP staining demonstrated that Y-Exos significantly suppressed osteoclast activation in both the subchondral bone and trabecular regions, while O-Exos showed no significant effect (Figure 7D, G(b-c)). Immunofluorescence analysis revealed that Y-Exos markedly increased OCN expression around the subchondral bone and trabecular regions and enhanced CD206 expression in the bone marrow of OVX mice (Figure 7E-F, H). In contrast, O-Exos did not significantly affect osteogenic activity or CD206 expression.

To further evaluate the effects of plasma-derived exosomes on immune regulation, bone marrow macrophage polarization was analyzed by flow cytometry. Neither Y-Exos nor O-Exos significantly altered the proportion of CD86⁺ M1 macrophages. However, Y-Exos significantly restored the decreased CD206⁺ macrophage population in OVX mice and increased the CD206⁺/CD86⁺ ratio, whereas O-Exos showed no significant regulatory effect on macrophage polarization (Figure 7I-J). Given that osteoporosis is associated with impaired bone marrow hematopoiesis^27^, ^28^ and that hematopoietic activity contributes to bone homeostasis through regulation of the bone marrow microenvironment^29^, we further examined hematopoietic cell populations after exosome treatment. Flow cytometry analysis showed that Y-Exos significantly increased the proportion of LSK hematopoietic progenitor cells and enhanced GMP differentiation, whereas O-Exos had no significant effects on hematopoietic cell differentiation (Figure 7K-L). These findings demonstrate that Y-Exos exert protective effects against OVX-induced bone loss by promoting bone formation, reducing osteoclast activation, enhancing M2 macrophage polarization, and partially restoring bone marrow hematopoietic activity.

To further investigate the molecular basis underlying the distinct biological effects of Y-Exos and O-Exos, proteomic mass spectrometry was performed to compare their protein compositions. PCA analysis showed clear separation between the Y-Exos and O-Exos groups, indicating distinct proteomic profiles (Figure 8A). Consistently, hierarchical clustering analysis revealed different expression patterns of proteins between the two groups (Figure 8B). Using the screening criteria of |log₂(FC)| > 1 and P < 0.05, a total of 128 differentially expressed proteins were identified, including 104 upregulated and 24 downregulated proteins in Y-Exos compared with O-Exos (Figure 8C). The top 10 proteins with the largest absolute log₂(FC) values were MAVS, S100A11, PLS3, IDE, FAM151A, FABP4, VCL, HPGD, YOD1, and ITGB2 (Figure 8D). Moreover, compared with O-Exos, Y-Exos showed a higher abundance of several proteins associated with bone homeostasis and osteogenic processes, including PRG4^30^, VCL^31^, SPP2^32^, THBS1^33^, and HSPA1A^34^. In addition, proteins involved in immunomodulatory processes and anti-inflammatory macrophage responses, such as CETP^35^, PRG4^36^, and NT5E^37^, were also enriched in Y-Exos. Conversely, several proteins with potential pro-inflammatory immunoregulatory functions, including FCGR3B^38^ and GSDMA^39^, were relatively increased in O-Exos (Figure 8E). These findings suggest that the functional difference between Y-Exos and O-Exos may result from broad remodeling of extracellular vesicle cargo composition rather than changes in a single protective molecule. GO and KEGG enrichment analyses were subsequently performed to characterize the potential functions of these differentially expressed proteins. Biological process analysis showed that these proteins were mainly associated with immune regulation, complement activation, cell-matrix adhesion, and other cellular processes (Figure 8F). Cellular component analysis indicated enrichment in extracellular vesicle-related structures, extracellular regions, membranes, and blood microparticles, consistent with the characteristics of exosome-derived proteins (Figure 8G). Molecular function analysis revealed enrichment in protein binding, receptor binding, and integrin-related functions (Figure 8H). KEGG analysis further indicated that the differentially expressed proteins were involved in pathways related to cytoskeletal regulation, cell adhesion, cell motility, and endocytosis (Figure 8I).

To identify functional proteins responsible for the distinct biological effects of Y-Exos and O-Exos, several differentially expressed proteins identified by proteomic analysis were further evaluated. Based on previous reports regarding their potential roles in inflammation and bone metabolism, PLS3, IDE, VCL, HPGD, YOD1, and ITGB2 were selected for functional screening. As shown in Figure S2-8 and Figure 9A-B, recombinant VCL, IDE, and YOD1 proteins exhibited regulatory effects on osteogenic differentiation and macrophage polarization under inflammatory conditions. Western blot analysis revealed that YOD1, but not VCL or IDE, showed a significant age-related decrease in plasma exosomes, with lower expression levels detected in exosomes from aged donors compared with young donors (Figure 9C). These findings suggest that reduced YOD1 abundance may contribute to the functional differences between Y-Exos and O-Exos. Flow cytometry was performed to further verify YOD1's regulatory effect on macrophage polarization. Under non-inflammatory conditions, 50 ng/mL YOD1 increased the proportion of CD206⁺ BMMs, whereas under LPS-induced inflammatory conditions, both 25 ng/mL and 50 ng/mL YOD1 increased CD206⁺ macrophage proportions. Notably, 50 ng/mL YOD1 also increased CD86⁺ macrophages under inflammatory stimulation, suggesting that YOD1 may regulate macrophage activation in a dose-dependent manner rather than inducing a purely polarized phenotype (Figure 9D-E). Figure 9F demonstrated that Y-Exos treatment significantly increased YOD1 levels in both BMMs and BMSCs, confirming the transfer of YOD1 cargo into recipient cells. Furthermore, conditioned medium from YOD1-treated LPS-stimulated BMMs was collected to evaluate the indirect effects of YOD1-mediated macrophage regulation on bone cells. YOD1 treatment significantly reversed the inhibitory effect of CM-LPS on osteogenesis and its promoting effect on osteoclast differentiation, as indicated by ALP/ARS and TRAP/F-actin staining analyses (Figure 9G-J). These findings indicate that YOD1 is an enriched functional component of Y-Exos and contributes to the regulation of macrophage polarization and bone cell differentiation.

To further explore the mechanism by which Y-Exos-delivered YOD1 regulates BMM differentiation, transcriptome sequencing was performed in LPS-stimulated BMMs with or without YOD1 treatment. PCA analysis demonstrated that the two sample groups exhibited distinct separation in their gene expression profiles (Figure 10A). Using the screening criteria of |log₂(FC)| > 1 and P < 0.05, 50 differentially expressed genes were identified, including 21 upregulated and 29 downregulated genes (Figure 10B). KEGG enrichment analysis revealed that these genes were mainly associated with Hippo and mTOR signaling pathways (Figure 10C). The effects of YOD1 and Y-Exos on Hippo/YAP signaling were subsequently examined. Under LPS stimulation, both YOD1 and Y-Exos increased ITCH expression, reduced LATS1 expression, decreased YAP1 phosphorylation, and promoted ARG1 expression while suppressing iNOS expression (Figure 10D, F). However, inhibition of YAP1 nuclear translocation by Verteporfin further enhanced the M2-associated effects of YOD1 and Y-Exos, characterized by increased ARG1 and reduced iNOS expression (Figure 10E, G). These findings suggest that YOD1 modulates Hippo/YAP signaling in BMMs, while YAP1 activity may contribute to pro-inflammatory macrophage activation under inflammatory stimulation. Transcriptomic analysis also indicated enrichment of the mTOR pathway. AKT/mTOR signaling was further investigated. YOD1 and Y-Exos significantly increased AKT and mTOR phosphorylation in LPS-stimulated BMMs (Figure 10H, I). Inhibition of AKT or mTOR activation using MK2206 or Rapamycin, respectively, abolished the YOD1- and Y-Exos-induced increase in ARG1 expression and restored iNOS expression (Figure 10J, L), indicating that AKT/mTOR signaling is required for the anti-inflammatory effects of YOD1 and Y-Exos. Moreover, Rapamycin partially reversed the inhibitory effects of YOD1- and Y-Exos-treated macrophage conditioned medium on osteoclast differentiation, as indicated by increased F-actin ring formation and NFATC1 expression (Figure 10N-P). In addition, the effects of YOD1 and Y-Exos on osteogenic differentiation were also investigated in BMSCs. YOD1 and Y-Exos modulated Hippo/YAP signaling in BMSCs, accompanied by increased expression of osteogenic markers RUNX2 and OCN (Figure 10K, M). Inhibition of YAP1 nuclear translocation by Verteporfin significantly attenuated the osteogenic-promoting effects of YOD1 and Y-Exos (Figure S9), indicating that YOD1/Y-Exos enhance osteogenic differentiation through regulation of Hippo/YAP signaling in BMSCs. To further determine whether YOD1 is necessary for the biological effects of Y-Exos, YOD1 expression in BMM and BMSC were knocked down by si-YOD1, with si-NC as the negative control. YOD1 knock-down significantly inhibited the ALP activity, decreased ITCH expression, increased LATS1 expression, increased YAP1 phosphorylation, and reduced RUNX2 and OCN expression. Y-Exos treatment partially rescued the osteogenic-inhibiting effects of si-YOD1 (Figure S9C-D, G, I). Moreover, Y-Exos treatment also partially reversed the si-YOD1-induced increase in CD86 expression, decreased CD206 expression, decreased mTOR phosphorylation, increased INOS expression, and decreased ARG1 expression in LPS-treated BMM (Figure S9E-F, H, J). These results demonstrate that YOD1 delivered by Y-Exos regulates macrophage polarization mainly through AKT/mTOR signaling, while modulating Hippo/YAP signaling in a cell-type-dependent manner to promote macrophage regulation and osteogenic differentiation.

## Discussion

Dysregulation of the osteoimmune microenvironment, especially proinflammatory macrophage polarization and subsequent disruption of bone homeostasis, constitutes an important pathological basis for the development of osteoporosis^40^. Recent studies have shown that young plasma and its derived exosomes exert anti-aging and anti-inflammatory effects, demonstrating therapeutic potential in multiple systemic diseases^41^-^43^. However, whether young plasma can regulate bone regeneration through remodeling of the osteoimmune microenvironment remains largely unknown. This study demonstrated that young plasma infusion alleviated systemic and bone marrow inflammation, enhanced osteogenic activity, and rescued bone loss in OVX mice. Mechanistically, plasma exosome depletion markedly attenuated these protective effects, identifying young plasma-derived exosomes as major mediators of its osteoprotective function. Further *in vivo* and *in vitro* studies revealed that Y-Exos restored bone homeostasis by promoting macrophage polarization toward an anti-inflammatory M2 phenotype, enhancing BMSC osteogenic differentiation, and improving the bone marrow microenvironment. Proteomic profiling and functional validation identified YOD1 as an important functional mediator enriched in Y-Exos. Mechanistically, Y-Exos-delivered YOD1 regulated distinct signaling pathways in different target cells, activating AKT/mTOR signaling to promote M2 polarization in BMMs and modulating Hippo/YAP signaling to enhance osteogenic differentiation in BMSCs. Therefore, this study provides evidence that young plasma and its exosomes ameliorate osteoporosis through osteoimmune microenvironment remodeling and identifies YOD1-containing exosomes as a potential therapeutic strategy for restoring bone homeostasis.

Young plasma and its components have emerged as promising strategies for counteracting age-related functional decline across multiple organs. Early studies using heterochronic parabiosis demonstrated that exposure of aged mice to a young circulatory environment could partially reverse tissue aging phenotypes. Conboy *et al*. showed that young blood exposure restored age-associated decline in Notch signaling, thereby improving the regenerative capacity of muscle satellite cells and hepatic progenitor cells in aged mice^44^. Subsequently, Villeda *et al*. reported that young blood circulation enhanced hippocampal synaptic plasticity and ameliorated age-related cognitive impairment by reversing molecular and functional alterations in the aging brain^25^. These findings indicated that circulating factors within young blood can systemically influence aging processes. With further mechanistic investigations, studies have identified specific proteins or components as the core mediators of the anti-inflammatory and anti-aging effects of young plasma. Gaudilliere *et al*. demonstrated that administration of plasma proteins from young donors modulated immune aging in older individuals by activating anti-inflammatory regulatory pathways and increasing beneficial immune cell subsets^43^. In addition, Chen *et al*. found that young plasma-derived exosomes exerted neuroprotective effects by inhibiting neural stem cell pyroptosis and reducing inflammatory responses through miR-16-5p-mediated suppression of NLRP3 inflammasome activation^42^. Together, these studies indicate that regulation of inflammatory responses and immune microenvironments represents a key mechanism through which young plasma and its components exert systemic protective effects. However, whether these rejuvenating factors can remodel the osteoimmune microenvironment and restore bone homeostasis during osteoporosis remains unclear.

In the present study, we demonstrated that young plasma infusion alleviated systemic and bone marrow inflammation, enhanced osteogenic activity, suppressed osteoclast activation, and rescued bone loss in OVX mice. Removal of exosomes markedly attenuated the therapeutic efficacy of plasma, indicating that plasma exosomes are the key effector components responsible for the immunomodulatory and bone-protective effects of young plasma. However, exosome depletion did not completely abolish the effects, suggesting the presence of other synergistic anti-anabolic and bone-promoting factors, such as soluble cytokines^45^, ^46^. Therefore, the bone-protective effects observed in Plasma-Y may not be solely attributed to its exosome components, but involve synergistic effects mediated by other soluble factors contained in young plasma. Aged plasma exerted no significant effect on osteogenic differentiation; conversely, it aggravated osteoclast activity in the subchondral bone region of OVX mice and caused bone loss, and exosome depletion alleviated this effect. These findings suggest that age-associated exosome alterations may contribute to the bone-destructive effects of aged plasma. Proteomic analysis further supported this hypothesis by revealing the enrichment of potentially pro-inflammatory or immunoregulatory proteins, including FCGR3B and GSDMA, in O-Exos^38^, ^39^. Therefore, the functional difference between Y-Exos and O-Exos may reflect age-associated remodeling of EV cargo, resulting in an altered immunoregulatory environment that possibly favors osteoclastogenesis. These findings are consistent with previous evidence showing that aging is associated with increased osteoclastogenic potential and that systemic age-related changes can contribute to enhanced bone resorption and deterioration of bone microarchitecture^47^, ^48^.

The present study further confirmed that Y-Exos can remodel the osteoimmune microenvironment both *in vivo* and *in vitro* by directing bone marrow macrophages toward M2 polarization and promoting the osteogenic differentiation of BMSCs. In contrast, O-Exos exhibited no immunomodulatory or osteogenic effects. Several studies have also reported the differential effects of Y-Exos and O-Exos on bone metabolism. For example, Y-Exos significantly enhanced the osteogenic differentiation and migration of BMSCs, whereas O-Exos exerted no obvious influence on BMSC osteogenic differentiation but instead promoted adipogenic differentiation of BMSCs and increased fatty infiltration in the femurs of OVX mice^49^. Another study reported that both Y-Exos and O-Exos promoted BMSC osteogenic differentiation and improved proximal femoral bone mass in OVX mice, although the stimulatory effect of O-Exos was markedly weaker than that of Y-Exos^50^. The effects of O-Exos may vary somewhat across studies due to differences in the old donors from which they are derived, but Y-Exos have been consistently shown to promote BMSC osteogenic differentiation and alleviate bone loss in osteoporosis. Notably, unlike previous reports focusing on the direct effects of exosomes on osteoblasts, the present study explored a novel mechanism underlying the therapeutic effect of Y-Exos in osteoporosis through osteoimmune regulation, thereby expanding understanding of the role of young plasma-derived exosomes in bone metabolism.

Previous studies have reported that Y-Exos deliver various miRNAs, such as miR-142-5p and miR-217-5p, to exert osteoprotective effects by regulating BMSC osteogenic differentiation and improving mitochondrial activity in aged organs and tissues^22^, ^23^, ^49^. However, the differences in protein components between Y-Exos and O-Exos, as well as their functions, remain unclear. Proteomic analysis and functional verification in this study confirmed that YOD1 was one of the key effector proteins responsible for the osteoprotective role of Y-Exos. YOD1 is a deubiquitinating enzyme regulating the HIPPO/YAP pathway through deubiquitinating and modifying ITCH to enhance its stability, thereby promoting LATS1 degradation, reducing LATS1-mediated phosphorylation of downstream YAP1, and enhancing YAP1/TAZ activity^51^. YAP1 plays a critical role in cellular mechanotransduction in bone, and increased nuclear translocation of YAP1 promotes osteogenic differentiation^52^. Accordingly, this study verified that YOD1 or Y-Exos increased YAP1 nuclear entry and promoted BMSC osteogenic differentiation. However, direct evidence linking YOD1 to macrophage polarization is limited. The HIPPO/YAP pathway has been reported to exert context-dependent effects on macrophage function. Some studies have shown that reduced YAP nuclear activity decreased the expression of M1-associated markers^53^, whereas increased YAP activation promoted the expression of IL-1β and iNOS in BMMs^54^. Conversely, inhibition of YAP nuclear translocation in M2 macrophages impaired their osteogenesis-promoting effects on co-cultured MC3T3 cells^55^. In the present study, verteporfin treatment inhibited YOD1-induced YAP1 nuclear translocation and reduced iNOS expression, suggesting that YAP1 may contribute to the pro-inflammatory response under our experimental conditions. Therefore, this YOD1-mediated YAP activation cannot fully explain the overall M2-polarizing function of YOD1 observed in this study. This discrepancy indicated that YOD1 might regulate macrophage polarization through multiple pathways. Consistently, the pathway analysis also identified mTOR signaling as another potential downstream mechanism involved in YOD1-mediated regulation of macrophage function. Previous studies have shown that activation of the AKT/mTOR pathway regulates macrophage M2 differentiation^56^, ^57^. This study confirmed that phosphorylation-mediated activation of AKT/mTOR was a key step for Y-Exos and YOD1 to drive BMM M2 phenotype differentiation. This finding indicates that YOD1 can exert regulatory effects via distinct mechanisms in different target cells. Besides, this study observed the dose-dependent effects of YOD1 on macrophage polarization in the *in vitro* test. We speculated that at a lower concentration (25 ng/mL), YOD1 activated anti-inflammatory pathways, such as mTOR-associated signaling, resulting in reduced expression of M1 markers. However, at a higher concentration (50 ng/mL), stronger activation of multiple signaling pathways, including both inflammatory and anti-inflammatory pathways, might occur simultaneously, leading to increased expression of both M1 and M2 polarization markers. Thus, the dose-dependent effects of YOD1 might reflect a balance between different signaling pathways, with the final macrophage phenotype determined by the relative strength of these regulatory signals. Further studies are required to clarify the precise molecular mechanisms underlying the dose-dependent regulation of macrophage polarization by YOD1.

It also deserves mention that although YOD1 was retained at a certain level in O-Exos, the overall changes in cargo composition may limit their capacity to reproduce the regenerative and immunomodulatory effects of Y-Exos. Besides, exosomal functions are also influenced by other molecular constituents, such as nucleic acids and lipids, which may undergo age-dependent changes. Future studies combining integrated omics approaches with targeted validation strategies will be necessary to define how age-related cargo remodeling of plasma-derived exosomes contributes to bone regeneration. Several additional candidates, including VCL and IDE, also showed potential regulatory effects on osteogenic and inflammatory processes, suggesting that Y-Exos exert their effects through a coordinated network of bioactive components rather than a single effector. Although YOD1 contributed to the biological effects of Y-Exos, it is unlikely to fully explain the regenerative capacity of Y-Exos. Nevertheless, the specific roles and interactions among these altered cargos require further investigation.

The present study found that the proportion of LSK cells in the bone marrow of OVX mice was decreased. Previous studies have shown that estrogen promotes the production of bone marrow hematopoietic cells and that estrogen levels regulate hematopoietic activity^58^. As an important component of the hematopoietic microenvironment, osteoblasts can also support hematopoietic stem cell generation by secreting cytokines^58^. In this study, ovariectomy led to decreased estrogen levels, reduced BMSC differentiation capacity, and increased bone marrow adipocyte infiltration, all of which may disrupt the microenvironment required for hematopoietic stem cell generation and exert a suppressive effect on hematopoiesis. In contrast, Y-Exos treatment significantly increased the proportions of LSK and GMP cells, which may be attributed to its ability to ameliorate the inflammatory bone marrow microenvironment, promote BMSC osteogenic differentiation, and reduce fat infiltration in the bone marrow cavity. Furthermore, the composition and differentiation of hematopoietic progenitors and myeloid cells, together with macrophage polarization, may reshape the local inflammatory environment and thereby influence subsequent bone regeneration^29^. Therefore, the changes in hematopoietic populations made by Y-Exos may subsequently contribute to a more favorable immune and bone microenvironment, potentially supporting bone formation and limiting excessive bone resorption. Nevertheless, whether the changes in LSK and GMP populations directly mediate the protective effects of Y-Exos against osteoporosis requires further investigation.

### Strength and limitation

This study has the following advantages over previous research: First, it revealed the mechanism underlying the therapeutic effect of young plasma exosomes on osteoporosis from the perspective of osteoimmunomodulation, distinct from previous studies focusing on direct effects on osteoblasts. Second, this study identified YOD1 as a key effector protein of Y-Exos, uncovering a novel functional molecule through which young plasma exosomes regulate bone metabolism. Third, this study elucidated the mechanism by which YOD1 partially mediated the effects of Y-Exos on regulating macrophage polarization and osteogenic differentiation via the mTOR/Hippo pathways, respectively, thereby synergistically improving bone homeostasis. These findings provide experimental evidence for the development of therapeutic strategies against osteoporosis based on young plasma exosomes, with potential for clinical translation. However, this study also has several limitations. For one, the composition of Y-Exos is complex, and numerous other effective components, such as additional proteins and nucleic acids beyond YOD1, remain to be identified and verified. In addition, this study investigated the therapeutic effect of Y-Exos in an OVX mouse model, and whether it applies to other types of osteoporosis, such as senile osteoporosis and glucocorticoid-induced osteoporosis, remains to be explored. Furthermore, although no obvious toxic or side effects of human-derived exosomes were observed in OVX mice, differences in immune compatibility in the xenogeneic model may have affected the results. Validation using humanized mouse models in future studies will enhance the persuasiveness of the findings.

## Conclusion

In summary, this study demonstrates that young plasma and exosomes derived from young plasma exert osteoimmunomodulatory effects. YOD1 partially mediated the effects of Y-Exos on activating the mTOR pathway in BMMs, thereby inducing M2 polarization, indirectly inhibiting osteoclast activation under inflammatory conditions, and relieving osteogenesis suppression. Meanwhile, Y-Exos activate the Hippo pathway in BMSCs to promote osteogenic differentiation. Ultimately, Y-Exos exert a synergistic osteoprotective effect through immunomodulation and enhanced osteogenesis. The findings of this study provide a novel theoretical basis for the development of therapeutic strategies against osteoporosis based on young plasma exosomes.

## Supplementary Material

Supplementary figures.

## Figures and Tables

**Figure 1:**
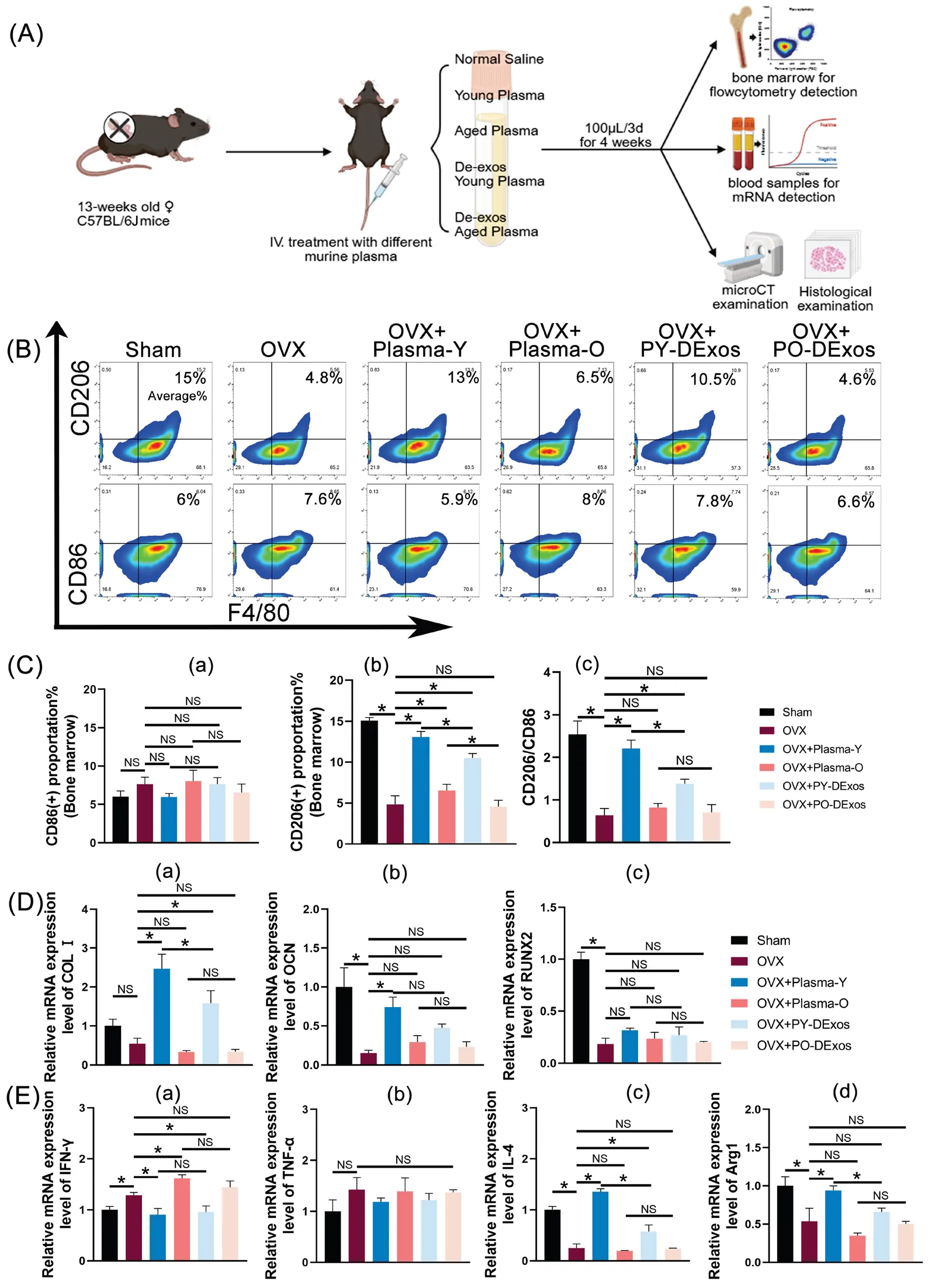
** Effects of young and aged plasma and exosome depletion on macrophage polarization and osteogenic and inflammatory markers in OVX mice.** (A) Schematic illustration of the experimental design. (Created in BioRender. Tu, W. (2026) https://BioRender.com/161nihb). (B) Representative flow cytometry plots showing bone marrow macrophage polarization in mice treated with sham operation (Sham), PBS (OVX), young plasma (Plasma-Y), aged plasma (Plasma-O), exosome-depleted young plasma (PY-DExos), or exosome-depleted aged plasma (PO-DExos). (C) Quantification of CD86⁺ macrophages (a), CD206⁺ macrophages (b), and the CD206⁺/CD86⁺ ratio (c), n=5. (D) qPCR analysis of peripheral blood osteogenic markers COL-I (a), OCN (b), and RUNX2 (c), n=3. (E) qPCR analysis of peripheral blood inflammatory and anti-inflammatory markers IFN-γ (a), TNF-α (b), IL-4 (c), and Arg1 (d), n=3. Data are presented as mean ± SD. *P < 0.05; NS, not significant.

**Figure 2:**
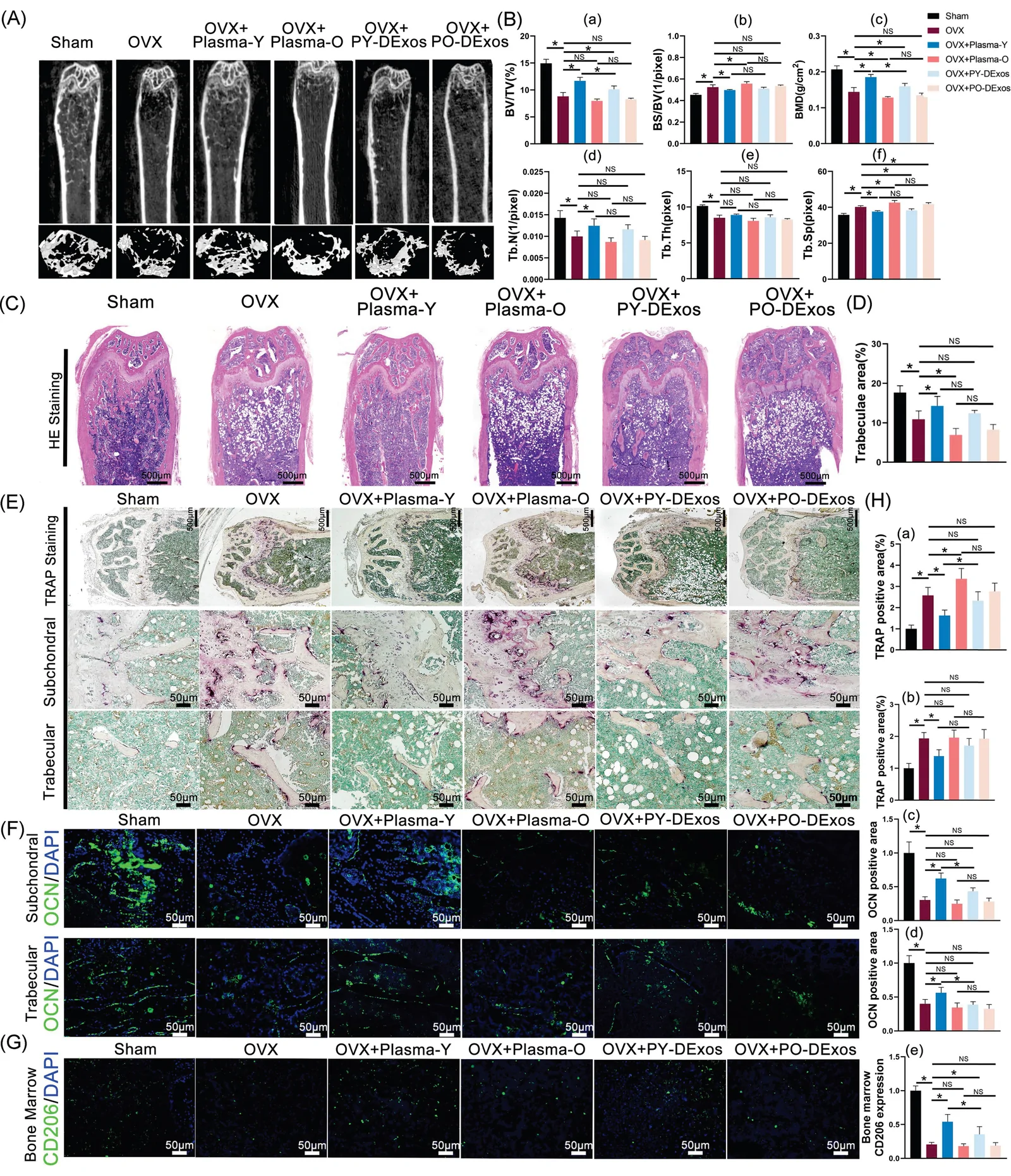
** Effects of different plasma treatments on femoral bone mass and histological changes in OVX mice.** (A) Representative coronal and three-dimensional micro-computed tomography (micro-CT) images of the proximal femur showing changes in bone mass. (B) Quantitative analyses of bone volume fraction (BV/TV), bone surface area-to-volume ratio (BS/BV), bone mineral density (BMD), trabecular number (Tb.N), trabecular thickness (Tb.Th), and trabecular separation (Tb.Sp), n=6. (C) Hematoxylin and eosin (HE) staining of the proximal femur (scale bar = 500 μm), n=6. (D) Representative changes in the number and distribution of trabecular bone. (E) Tartrate-resistant acid phosphatase (TRAP) staining showing osteoclast activation in the subchondral bone and trabecular regions of the proximal femur (scale bar = 50 μm), n=6. (F) Immunofluorescence staining showing the expression of the osteogenic protein OCN in the subchondral bone and trabecular regions of the proximal femur, and (G) the expression of the M2 macrophage marker CD206 in the bone marrow of the proximal femur (scale bar = 50 μm), n=6. (H) Quantification of TRAP-positive area in the subchondral bone (a) and trabecular regions (b), OCN-positive area in the subchondral bone (c) and trabecular regions (d), and CD206-positive area in the bone marrow (e). Data are presented as mean ± standard deviation (SD). *P < 0.05; NS, no significant difference.

**Figure 3:**
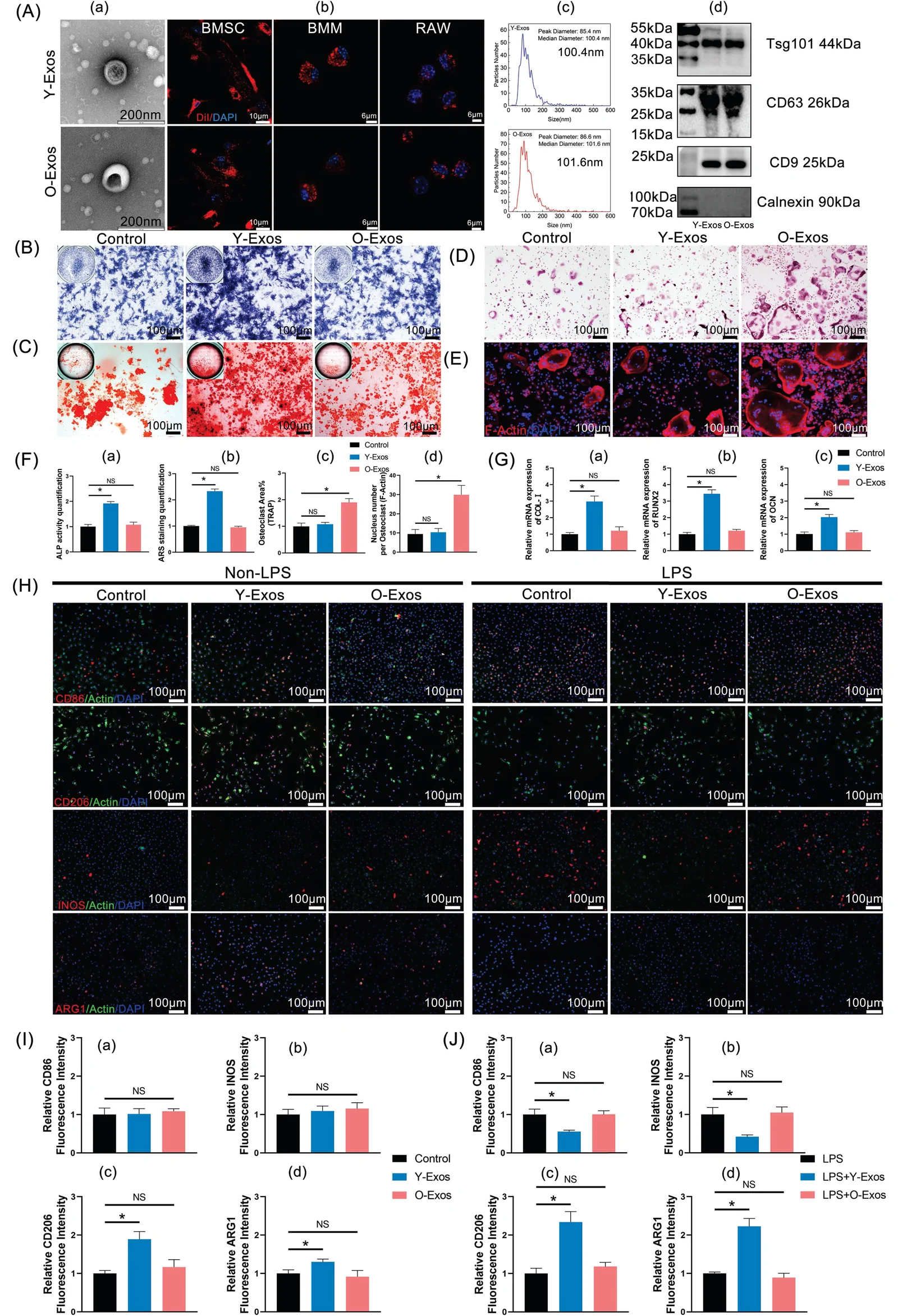
** Isolation and functional characterization of plasma-derived exosomes.** (A) Characterization of young plasma-derived exosomes (Y-Exos) and aged plasma-derived exosomes (O-Exos): (a) morphology observed by scanning electron microscopy (SEM) (scale bar = 200 nm); (b) uptake of Y-Exos and O-Exos by bone marrow-derived mesenchymal stem cells (BMSCs), bone marrow-derived macrophages (BMMs), and RAW264.7 cells (scale bars = 6 μm and 10 μm); (c) particle size distribution determined by nanoparticle tracking analysis (NTA); (d) expression of exosomal markers TSG101, CD63, and CD9 detected by Western blotting. (B-C) ALP and ARS staining evaluating the effects of Y-Exos and O-Exos on osteogenic differentiation of BMSCs (scale bar = 100 μm), n=3. (D-E) TRAP and F-actin ring staining assessing osteoclast differentiation of BMMs (scale bar = 100 μm), n=5. (F) Quantification of ALP activity (a), ARS staining (b), osteoclast area (c), and osteoclast nuclei number (d). (G) qPCR analysis of osteogenic genes COL-I, RUNX2, and OCN in BMSCs treated with Y-Exos or O-Exos, n=3. (H) Immunofluorescence staining of macrophage polarization markers CD86, CD206, iNOS, and ARG1 in BMMs under non-inflammatory and LPS-induced inflammatory conditions. Target proteins were labeled red, cytoskeletons were labeled green, and nuclei were counterstained with DAPI (scale bar = 100 μm), n=4. (I-J) Quantification of fluorescence intensity under non-LPS (I) and LPS-stimulated (J) conditions. Data are presented as mean ± SD. *P < 0.05; NS, no significant difference.

**Figure 4:**
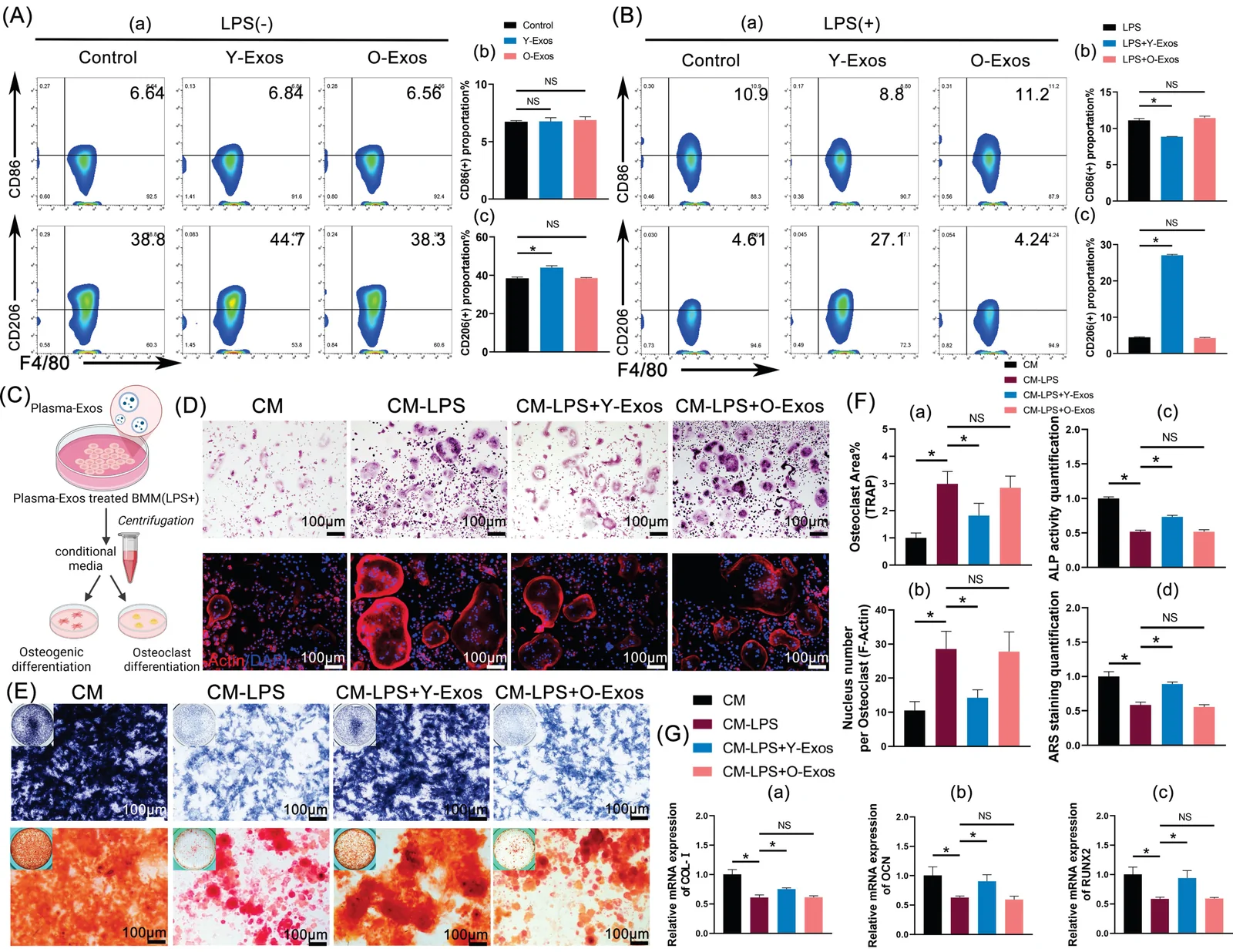
** Plasma-derived exosomes indirectly regulate osteogenic and osteoclastic differentiation by modulating macrophage polarization.** (A-B) Flow cytometric analysis of the effects of plasma-derived exosomes on macrophage polarization under non-LPS (A) and LPS-induced inflammatory conditions (B). Quantification of CD86⁺ and CD206⁺ macrophage ratios is shown, n=3. (C) Schematic illustration of the intervention of BMM-conditioned medium in osteogenic and osteoclastic differentiation assays. (Created in BioRender. Tu, W. (2026) https://BioRender.com/161nihb). (D) TRAP and F-actin ring staining evaluating the effects of conditioned medium on osteoclast differentiation (scale bar = 100 μm), n=5. (E) ALP and ARS staining assessing the effects of conditioned medium on osteogenic differentiation (scale bar = 100 μm), n=3. (F) Quantification of osteoclast differentiation area (a), osteoclast nuclei number (b), ALP activity (c), and ARS staining (d). (G) qPCR analysis of osteogenic genes COL-I, OCN, and RUNX2 in BMSCs treated with conditioned medium, n=3. Data are presented as mean ± SD. *P < 0.05; NS, no significant difference.

**Figure 5:**
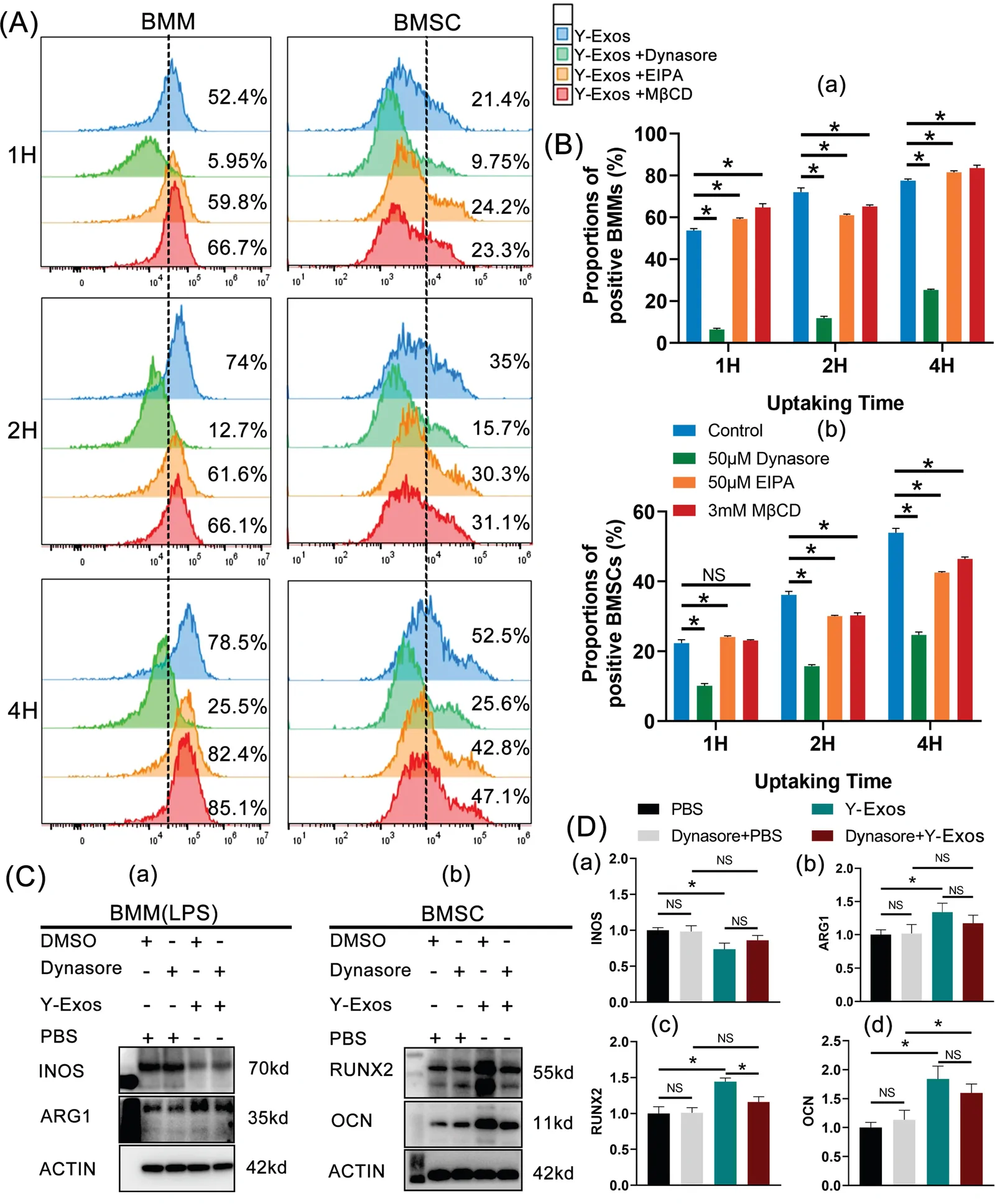
** Investigation of the uptake mechanism of plasma exosomes.** (A) Flow cytometric analysis of DiD-labeled Y-Exos uptake by BMMs and BMSCs after treatment with the endocytosis inhibitors Dynasore, EIPA, and MβCD at different time points (1 h, 2 h, and 4 h), n=3. (B) Quantification of DiD-positive cells in BMMs (a) and BMSCs (b) after different inhibitor treatments. (C) Western blot analysis of the effects of Dynasore-mediated inhibition of Y-Exos uptake on BMM polarization and BMSC osteogenic differentiation. (D) Quantification of iNOS, ARG1, RUNX2, and OCN protein expression, n=3. Protein expression was normalized to ACTIN and calibrated against the PBS group. Data are presented as mean ± SD. *P < 0.05; NS, no significant difference.

**Figure 6:**
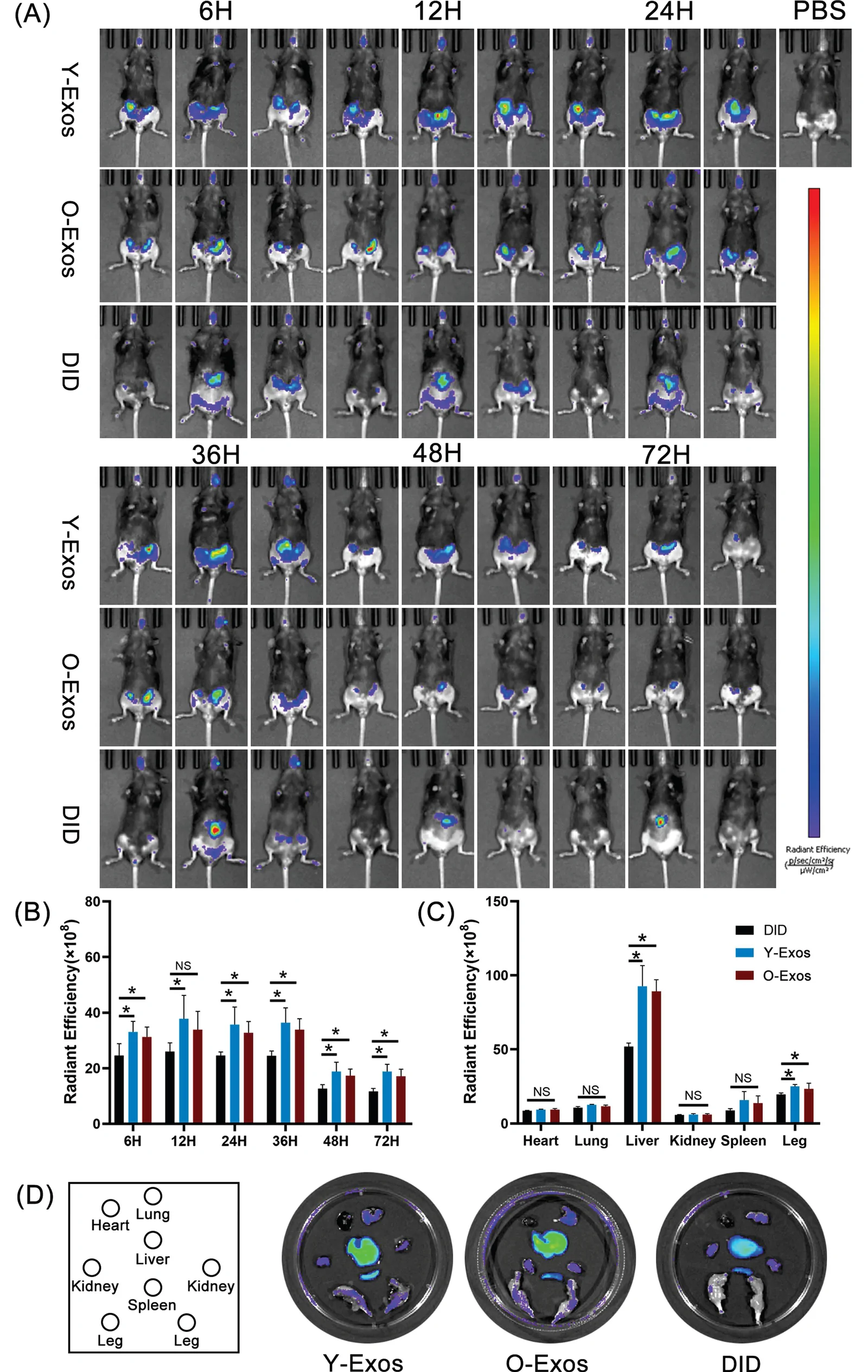
***In vivo* distribution of plasma-derived exosomes.** (A) *In vivo* fluorescence imaging of DiD-labeled Y-Exos and O-Exos after tail vein injection at different time points (6 h, 12 h, 24 h, 36 h, 48 h, and 72 h). (B) Quantification of femoral fluorescence intensity at each time point, n=5. (C-D) *Ex vivo* fluorescence imaging and quantification of major organs and isolated femurs at 72 h after injection, n=5. Data are presented as mean ± SD. *P < 0.05; NS, no significant difference.

**Figure 7:**
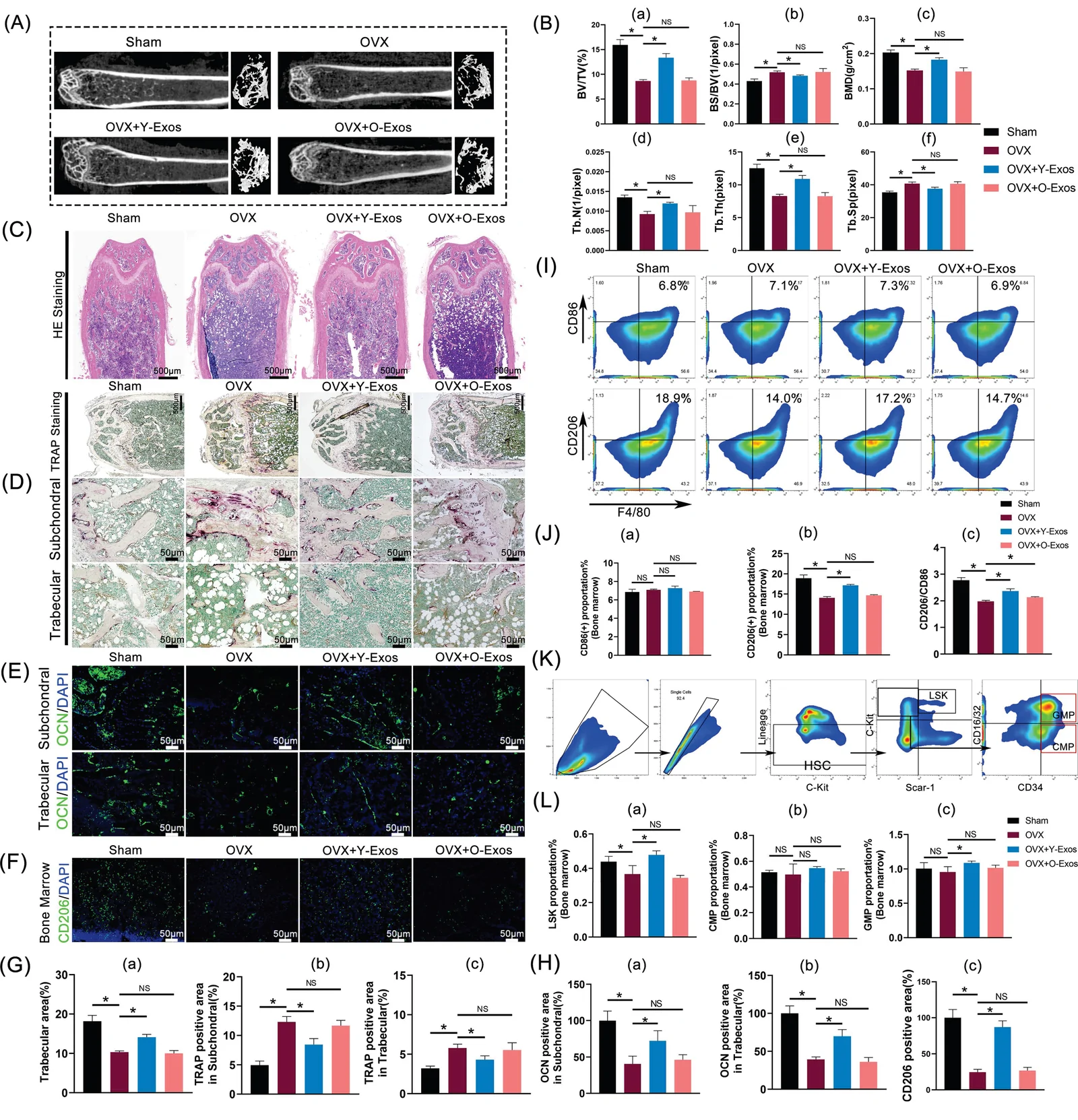
***In vivo* therapeutic effects of plasma-derived exosomes in OVX mice.** (A) Representative micro-CT images of the femur after Y-Exos or O-Exos treatment. (B) Quantification of bone parameters, including BV/TV (a), BS/BV (b), BMD (c), Tb. N (d), Tb.Th (e), and Tb.Sp (f), n=6. (C) HE staining showing trabecular bone distribution in the proximal femur (scale bar = 500 μm), n = 6. (D) TRAP staining showing osteoclast activation in the subchondral bone and trabecular regions (scale bar = 50 μm), n = 6. (E-F) Immunofluorescence staining of OCN expression in the proximal femur and CD206 expression in bone marrow (scale bar = 50 μm), n=6. (G) Quantification of trabecular bone area (a), TRAP-positive area in the subchondral bone (b), and trabecular region (c). (H) Quantification of OCN-positive area in the subchondral bone (a) and trabecular region (b), and CD206-positive area in bone marrow (c). (I-J) Flow cytometric analysis and quantification of bone marrow macrophage polarization, including CD86⁺ macrophages (a), CD206⁺ macrophages (b), and CD206⁺/CD86⁺ ratio (c), n=5. (K-L) Flow cytometric analysis and quantification of bone marrow hematopoietic cell populations, including LSK cells (a), common myeloid progenitors (CMP) (b), and granulocyte-monocyte progenitors (GMP) (c), n=6. Data are presented as mean ± SD. *P < 0.05; NS, no significant difference.

**Figure 8:**
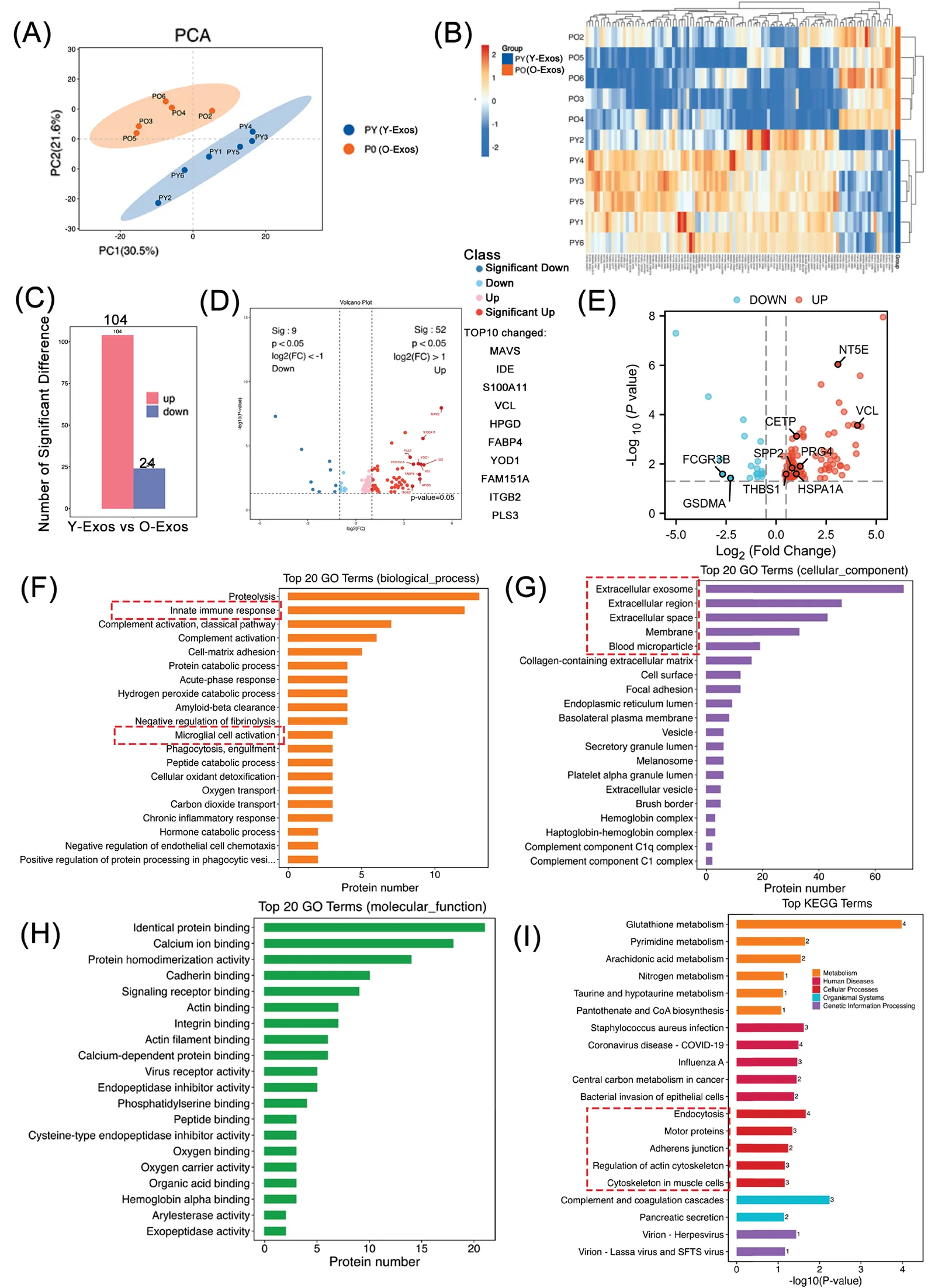
** Proteomic sequencing analysis of differentially expressed protein components in Y-Exos and O-Exos.** (A) Principal component analysis (PCA) based on proteomic mass spectrometry data. (B) Heatmap showing hierarchical clustering of differentially expressed proteins. (C) Number of upregulated and downregulated proteins identified between Y-Exos and O-Exos. (D) Volcano plot showing significantly differentially expressed proteins (P < 0.05, |log₂(FC)| > 1). Those associated with bone homeostasis and immune regulation were shown in (E). (F-I) Functional enrichment analyses of differentially expressed proteins, including Gene Ontology (GO) analysis of biological processes (F), cellular components (G), molecular functions (H), and Kyoto Encyclopedia of Genes and Genomes (KEGG) pathway analysis (I). Data are presented as mean ± SD. *P < 0.05; NS, no significant difference.

**Figure 9:**
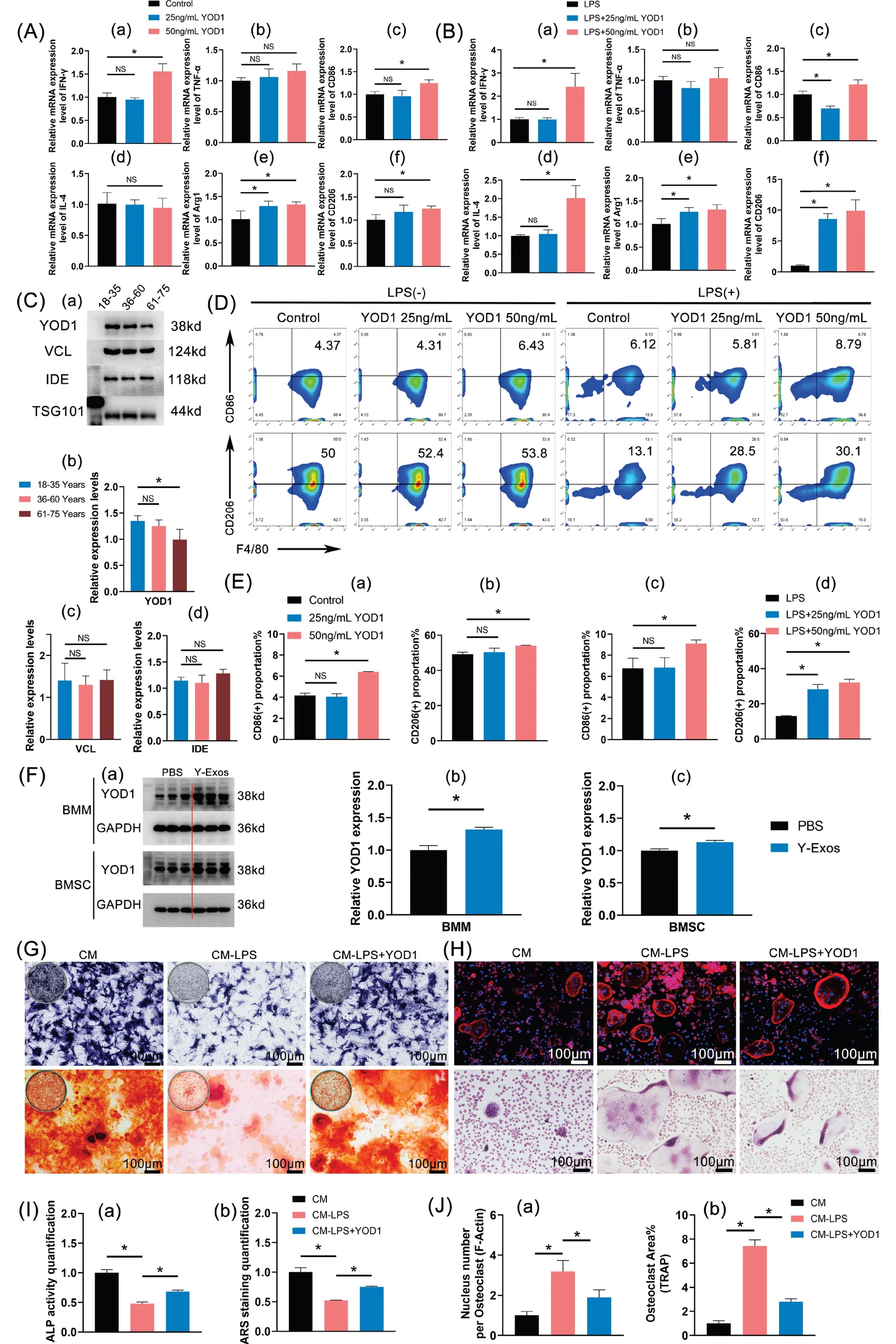
** Experimental validation of YOD1, a candidate protein enriched in Y-Exos, in regulating osteogenic differentiation and macrophage polarization.** (A) qPCR analysis of the effects of different concentrations of YOD1 (25 ng/mL and 50 ng/mL) on BMM polarization-related genes under non-inflammatory conditions, including TNF-α, IFN-γ, CD86, IL-4, Arg1, and CD206, n = 4. (B) Corresponding effects under LPS-induced inflammatory conditions, n = 4. (C) Western blotting analysis of YOD1, VCL, and IDE protein expression levels in plasma exosomes from donors of different ages, with TSG101 as the loading control, n = 3. (D-E) Flow cytometric analysis and quantification of CD86⁺ and CD206⁺ BMMs after YOD1 treatment under non-LPS and LPS conditions, n=3. (F) Western blotting analysis of YOD1 expression in BMMs and BMSCs after Y-Exos treatment, with GAPDH as the loading control, n = 3. (G) ALP staining and ARS staining evaluating the effect of YOD1 on BMSC osteogenic differentiation (scale bar = 100 μm), n = 3. (H) TRAP staining and F-actin ring staining evaluating the effect of YOD1 on BMM osteoclast differentiation (scale bar = 100 μm), n = 4. (I-J) Quantification of osteogenic differentiation (I: ALP activity and ARS staining) and osteoclast differentiation (J: osteoclast nuclei number and osteoclast area). Data are presented as mean ± SD. *P < 0.05; NS, no significant difference.

**Figure 10:**
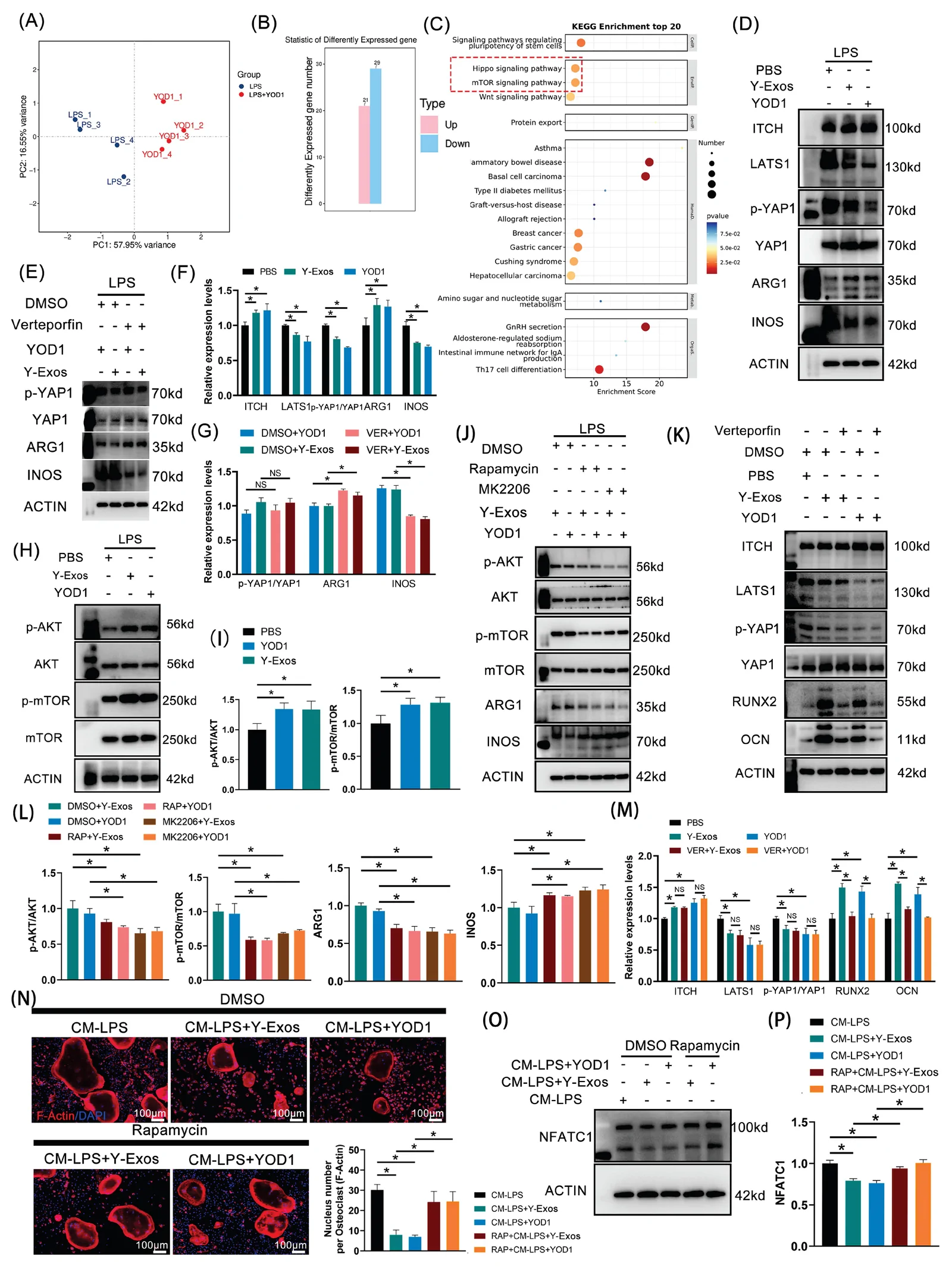
** Mechanistic analysis of YOD1-mediated regulation by Y-Exos in macrophage polarization and osteogenic differentiation.** (A) PCA analysis of transcriptomic profiles of LPS-stimulated BMMs treated with or without YOD1. (B) Number of differentially expressed genes identified after YOD1 treatment (P < 0.05, |log₂(FC)| > 1). (C) KEGG pathway enrichment analysis of differentially expressed genes. (D) Western blot analysis of Hippo/YAP signaling-related proteins and macrophage polarization markers in BMMs treated with YOD1 or Y-Exos under LPS stimulation. (E) Effects of YAP1 nuclear translocation inhibition by Verteporfin on ARG1 and iNOS expression in BMMs. (F-G) Quantification of protein expression in (D-E). (H) Western blot analysis of AKT/mTOR signaling-related proteins in BMMs after YOD1 or Y-Exos treatment. (I) Quantification of AKT/mTOR-related protein expression. (J) Effects of AKT and mTOR inhibition by MK2206 and Rapamycin on macrophage polarization-related proteins. (K) Western blot analysis of Hippo/YAP signaling-related proteins and osteogenic markers in BMSCs after YOD1 or Y-Exos treatment during osteogenic induction. (L-M) Quantification of protein expression in (J-K). (N) F-actin ring staining showing the effects of Rapamycin on the indirect regulation of osteoclast differentiation by YOD1 and Y-Exos (scale bar = 100 μm). (O-P) Western blot analysis and quantification of NFATC1 expression after Rapamycin treatment. Data are presented as mean ± SD. *P < 0.05; NS, no significant difference.

**Table 1:** Primer sequences

Primer	Sequences
GAPDH-F	5'-GGTTGTCTCCTGCGACTTCA-3'
GAPDH-R	5'-TGGTCCAGGGTTTCTTACTCC-3'
TNF-α-F	5'-AGCCGATGGGTTGTACCTTG-3'
TNF-α-R	5'-ATAGCAAATCGGCTGACGGT-3'
IFN-γ-F	5'-ATGAACGCTACACACTGCATC-3'
IFN-γ-R	5'-CCATCCTTTTGCCAGTTCCTC-3'
CD86-F	5'-TCTCCACGGAAACAGCATCT-3'
CD86-R	5'-CTTACGGAAGCACCCACGAT-3'
ARG1-F	5'-AAGAATGGAAGAGTCAGTGTGG-3'
ARG1-R	5'-GGGAGTGTTGATGTCAGTGTG-3'
IL4-F	5'-CTGTCTGATTTTGCTGTTGGTG-3'
IL4-R	5'-GATGTAGTCAGAGAAGCAGGTG-3'
CD206-F	5'-CCTATGAAAATTGGGCTTACGG-3'
CD206-R	5'-CTGACAAATCCAGTTGTTGAGG-3'
Col1-F	5'-CTGGCGGTTCAGGTCCAAT-3'
Col1-R	5'-CCACGTCTCACCATTGGGG-3'
Runx2-F	5'-ATGCTTCATTCGCCTCACAAA-3'
Runx2-R	5'-GCACTCACTGACTCGGTTGG-3'
OCN-F	5'-CTGACCTCACAGATCCCAAGC-3'
OCN-R	5'-TGGTCTGATAGCTCGTCACAAG-3'

## Data Availability

The data supporting the conclusions of this article are included within the article and its additional files.
