# Supplementary material for: Young plasma-derived exosomes deliver YOD1 to induce M2 polarization and ameliorate osteoporosis via mTOR signaling pathways
Source: Int J Biol Sci. 2026 Sep 18;22(15):8364–89. doi: 10.7150/ijbs.138620 (PMC13685755; doi:10.7150/ijbs.138620)
Supplement: Supplementary file 1 [file ijbsv22p8364s1.pdf]

Supplementary Figures

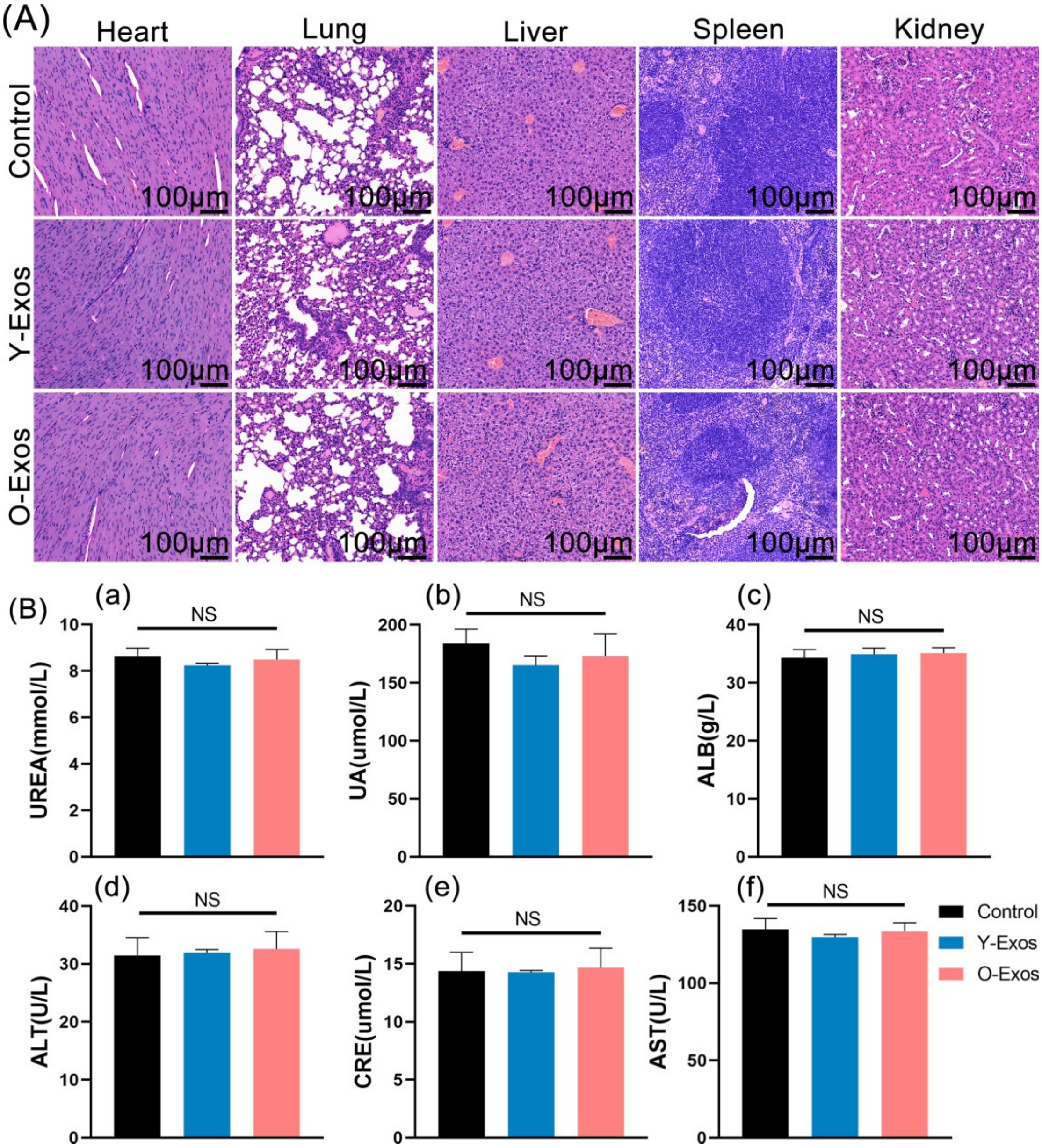

Figure S1: Detection of Y-Exos-induced systemic toxicity in OVX mice. (A) Histological morphology of major organs, including Heart, Lung, Liver, Spleen, and Kidney, after Y-Exos treatment was observed by HE staining. (B) Measurements of biochemical markers, including urea (UREA), uric acid (UA), albumin (ALB), alanine aminotransferase (ALT), creatinine (CRE), and aspartate aminotransferase (AST) after Y-Exos treatment in fracture mice, n = 4. All data are presented as mean ± standard deviation (SD). *P < 0.05; NS, no significant difference.

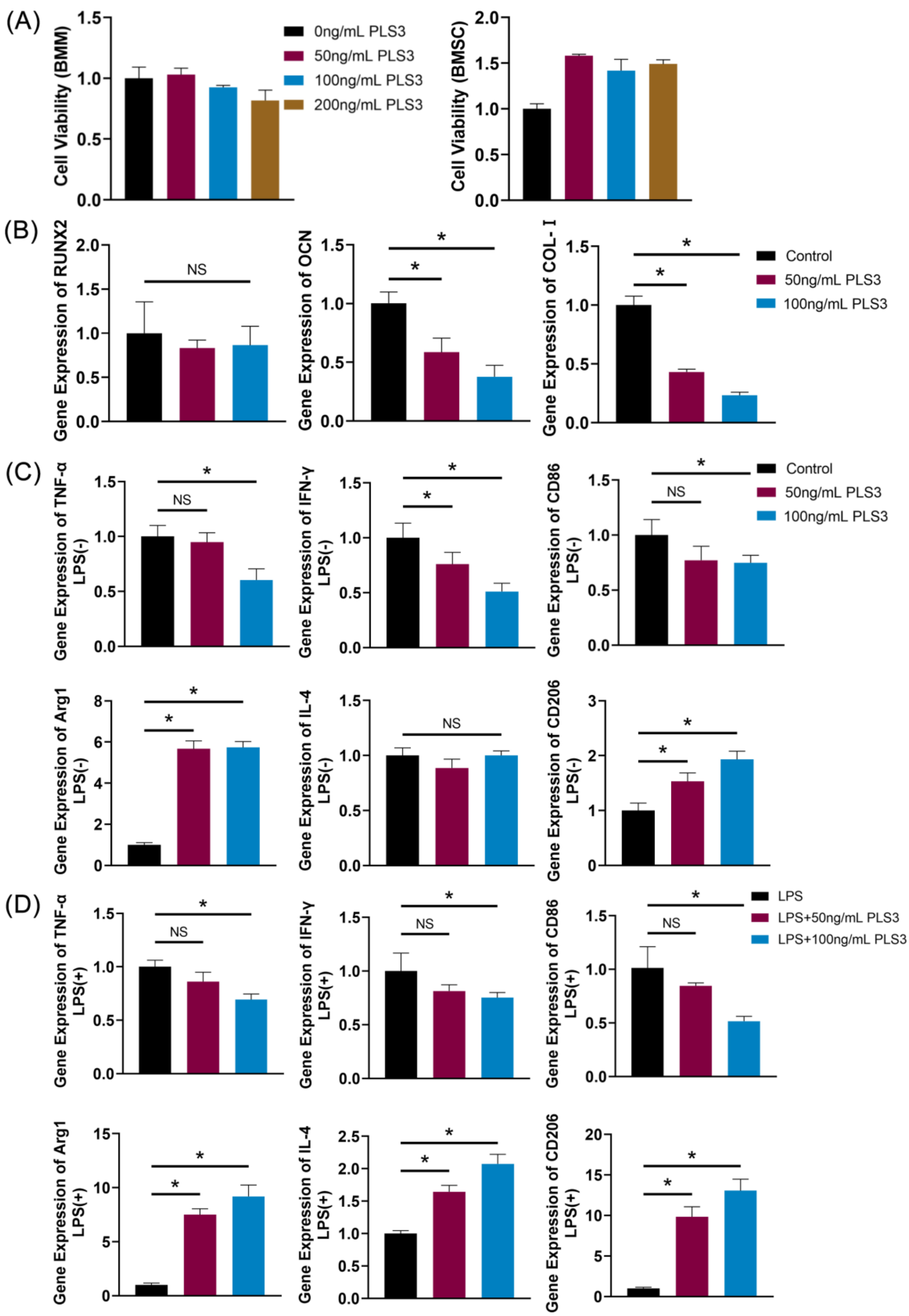

Figure S2. In vitro detection of the regulatory effects of PLS3 protein on macrophage polarization. (A) The CCK8 assay was used to measure the cell viability of BMM and BMSC after treatment with different concentrations of PLS3, n = 3. (B) qPCR was used to detect the expression of osteogenic genes (RUNX2, OCN, and COL-

I) after PLS3 treatment, n = 4. (C, D) qPCR was used to detect the expression of macrophage polarization genes (IFN-γ, TNF-α, CD86, ARG1, IL-4, and CD206) after PLS3 treatment under (C) LPS-free conditions or (D) with LPS treatment, n = 4. All data are presented as mean ± standard deviation (SD). *P < 0.05; NS, no significant difference.

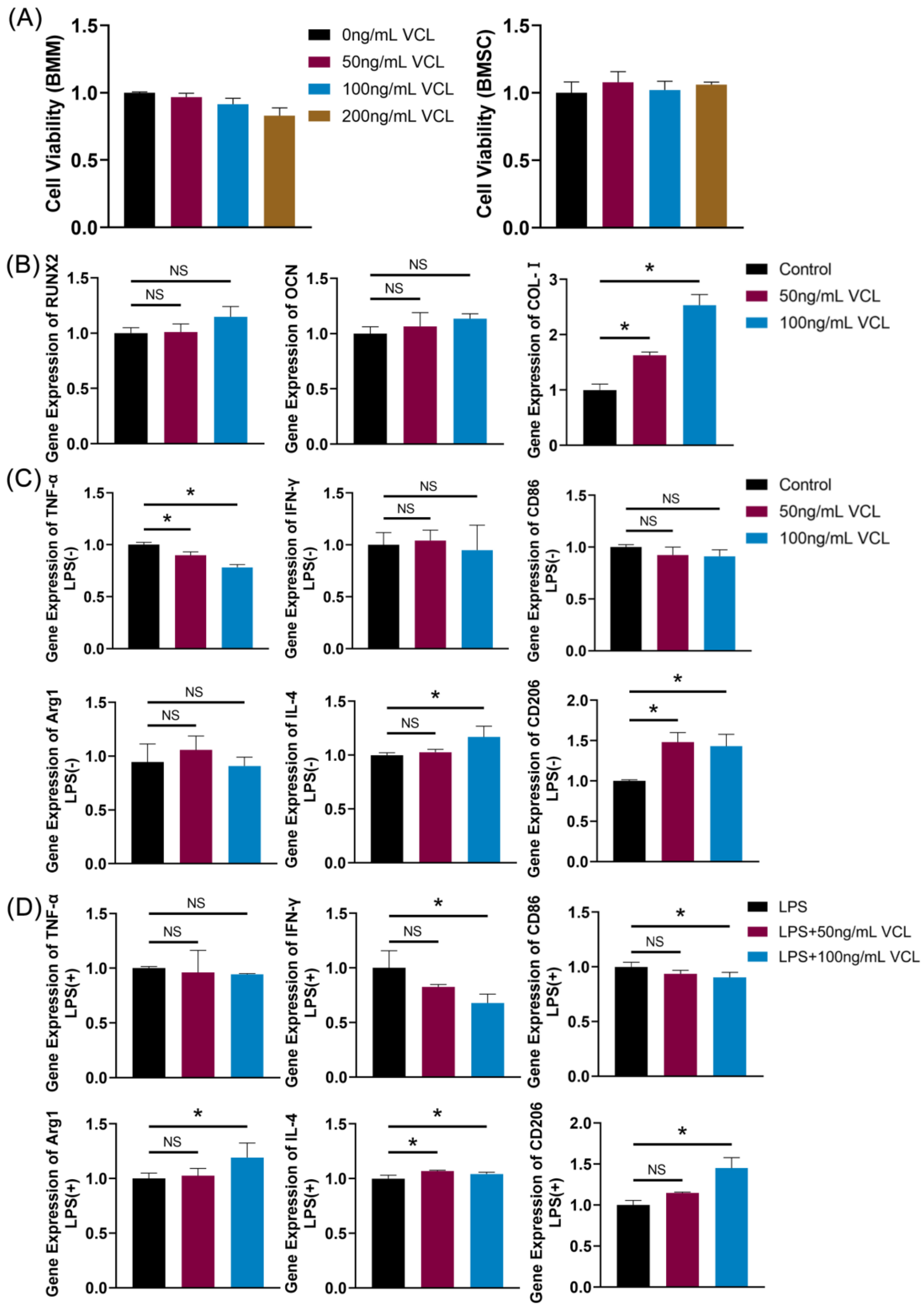

Figure S3. In vitro detection of the regulatory effects of VCL protein on macrophage polarization. (A) The CCK-8 assay was used to measure the viability of BMM and BMSC after treatment with varying concentrations of VCL, n = 3. (B) qPCR was used to detect the expression of osteogenic genes (RUNX2, OCN, and COL-I) after VCL treatment, n = 4. (C, D) qPCR was used to detect the expression of macrophage polarization genes (IFN-γ, TNF-α, CD86, ARG1, IL-4, and CD206) after VCL treatment under (C) LPS-free conditions or (D) with LPS treatment, n = 4. All data are presented as mean ± standard deviation (SD). *P < 0.05; NS, no significant difference.

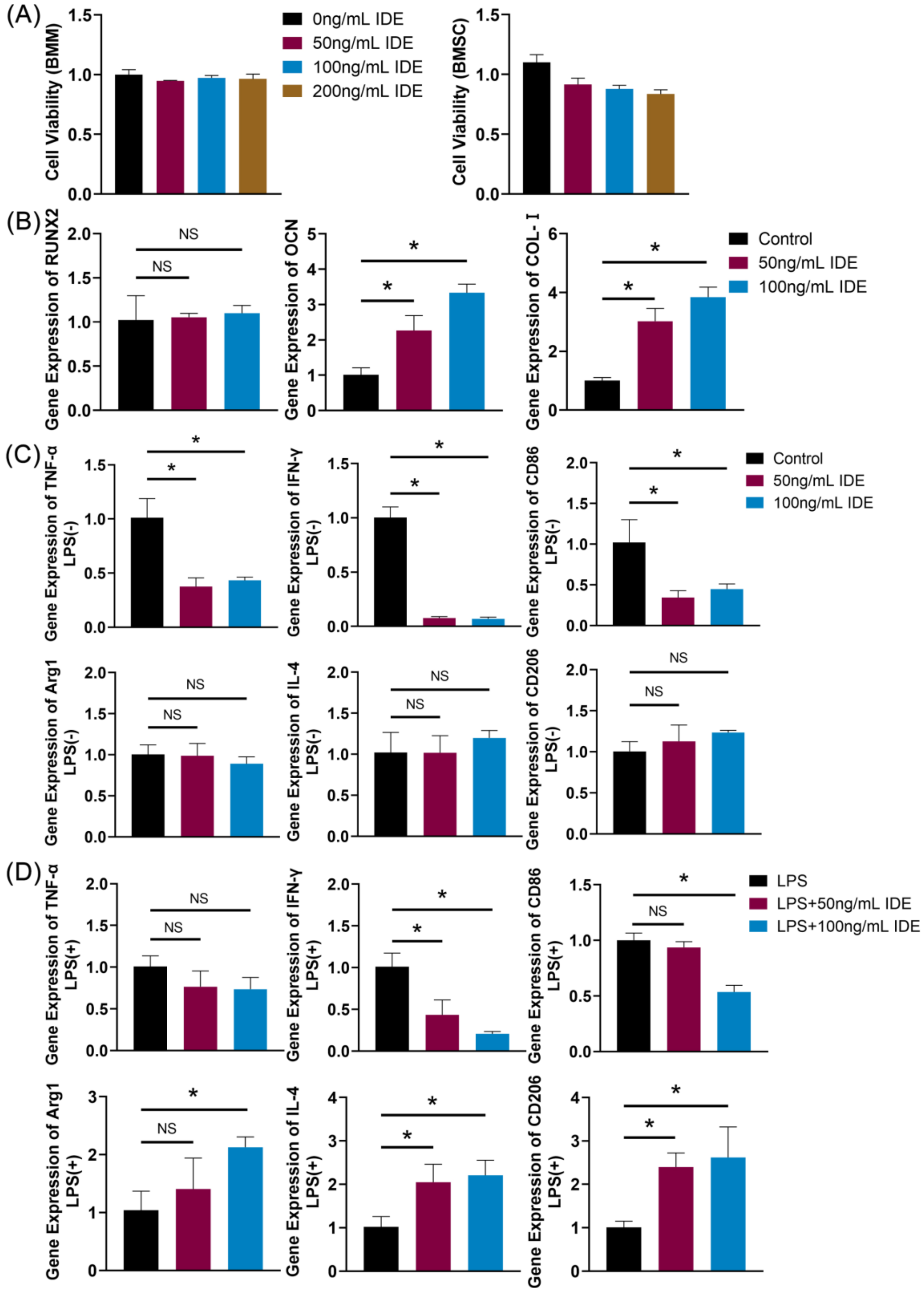

Figure S4. In vitro detection of the regulatory effects of IDE protein on macrophage polarization. (A) The CCK-8 assay was used to measure the viability of BMM and BMSC after treatment with varying concentrations of IDE, n = 3. (B) qPCR was used to detect the expression of osteogenic genes (RUNX2, OCN, and COL-I) after IDE treatment, n = 4. (C, D) qPCR was used to detect the expression of macrophage

polarization genes (IFN-γ, TNF-α, CD86, ARG1, IL-4, and CD206) after IDE treatment under (C) LPS-free conditions or (D) with LPS treatment, n = 4. All data are presented as mean ± standard deviation (SD). *P < 0.05; NS, no significant difference.

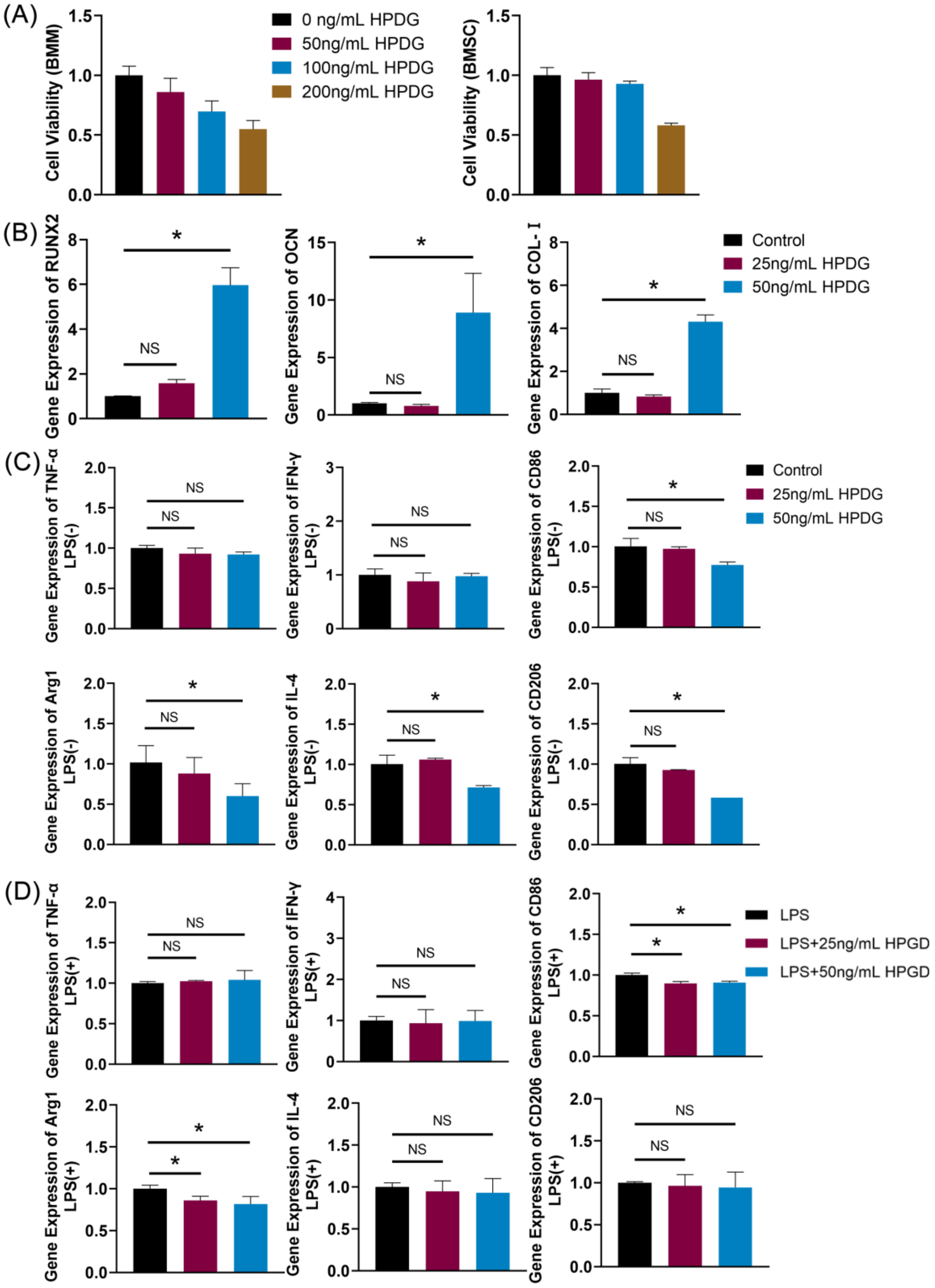

Figure S5. In vitro detection of the regulatory effects of HPGD protein on macrophage polarization. (A) The CCK8 assay was used to measure the cell viability

of BMM and BMSC after treatment with different concentrations of HPGD, n = 3. (B) qPCR was used to detect the expression of osteogenic genes (RUNX2, OCN, and COL-I) after HPGD treatment, n = 4. (C, D) qPCR was used to detect the expression of macrophage polarization genes (IFN-γ, TNF-α, CD86, ARG1, IL-4, and CD206) after HPGD treatment under (C) LPS-free conditions or (D) LPS-treated conditions, n = 4. All data are presented as mean ± standard deviation (SD). *P < 0.05; NS, no significant difference.

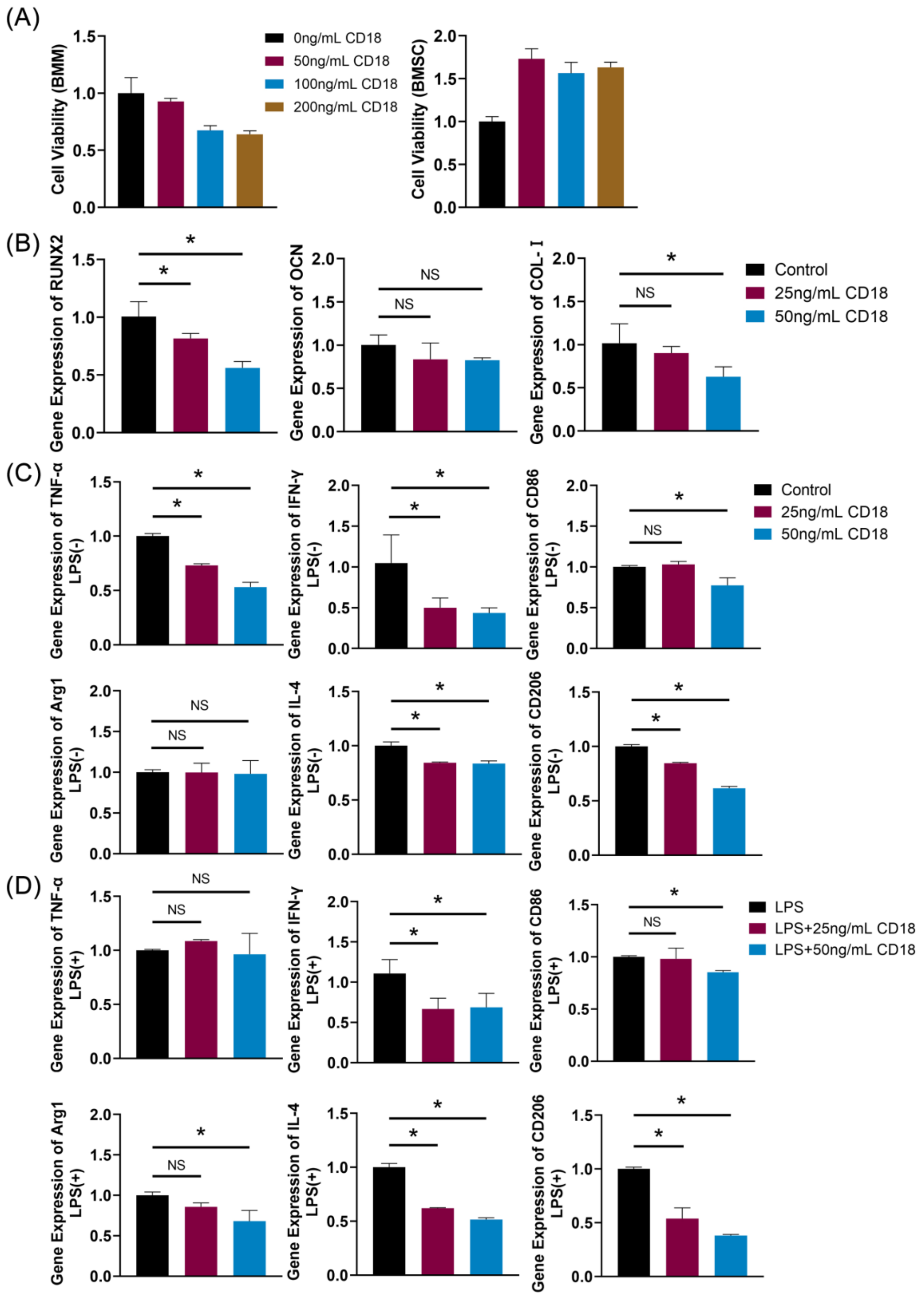

Figure S6. In vitro detection of the regulatory effects of CD18 protein on macrophage polarization. (A) The CCK8 assay was used to measure the cell viability of BMM and BMSC after treatment with different concentrations of CD18, n = 3. (B) qPCR was used to detect the expression of osteogenic genes (RUNX2, OCN, and COL-I) after CD18 treatment, n = 4. (C, D) qPCR was used to detect the expression of

macrophage polarization genes (IFN-γ, TNF-α, CD86, ARG1, IL-4, and CD206) after CD18 treatment under (C) LPS-free conditions or (D) with LPS treatment, n = 4. All data are presented as mean ± standard deviation (SD). *P < 0.05; NS, no significant difference.

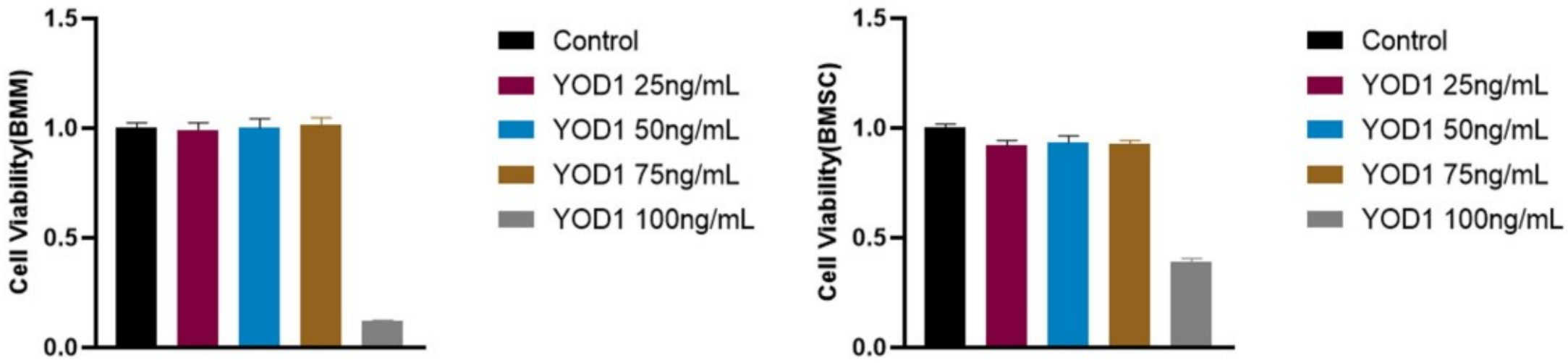

Figure S7. The CCK-8 assay was used to measure the viability of BMM and BMSC after treatment with varying concentrations of YOD1, n = 3. All data are presented as mean ± standard deviation (SD). *P < 0.05; NS, no significant difference.

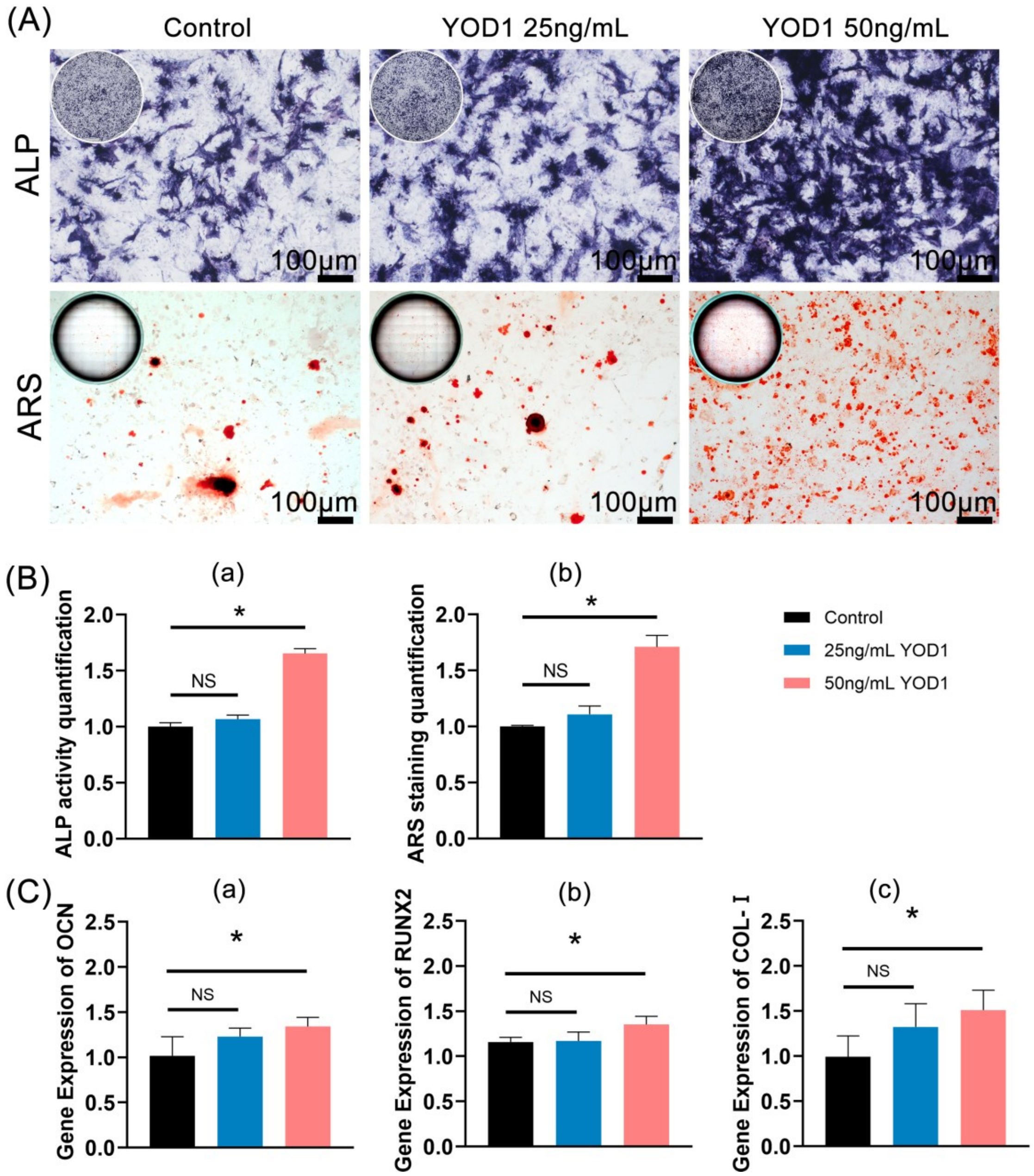

Figure S8. In vitro detection of the regulatory effects of YOD1 protein on osteogenesis. (A) ALP staining and ARS staining for evaluating the effects of YOD1 on osteogenic differentiation of BMSC (scale bar = 100 μm) , n = 3. (B) (a) Quantitative analysis of ALP activity; (b) Quantification of ARS staining. (C) qPCR analysis of the effects of YOD1 on the expression of osteogenic differentiation genes (a) OCN, (b) RUNX2, and (c) COL-I in BMSC, n = 4. All data are presented as mean ± standard deviation (SD). *P < 0.05; NS, no significant difference.

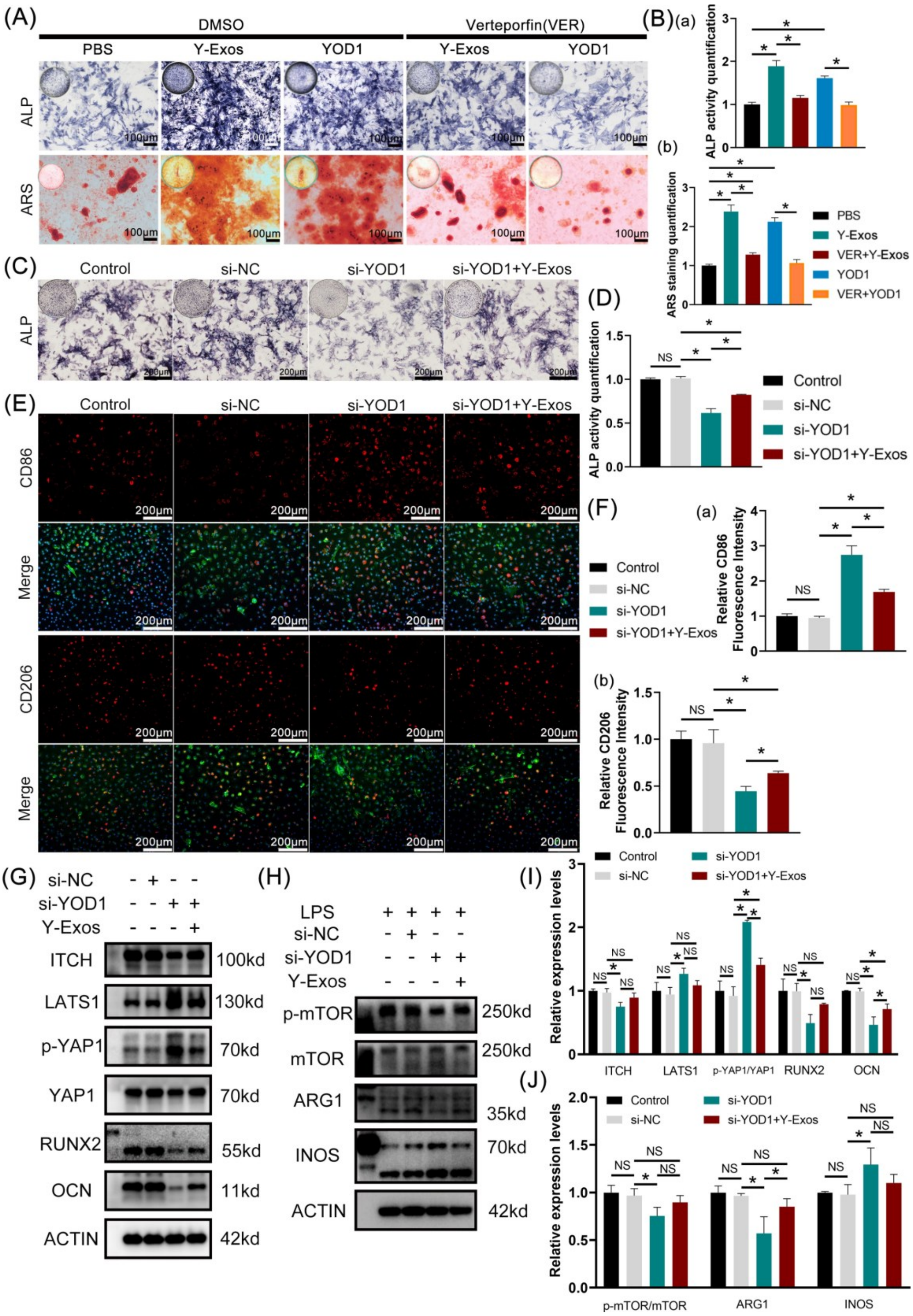

Figure S9. Verification of YOD1 function in the regulatory effects of Y-Exos on BMSC and BMM. (A) ALP and ARS staining to assess the impact of YAP1 blockade on the osteogenic-promoting effects of Y-Exos and YOD1 in BMSCs (scale bar = 100 μm), n = 3. (B) (a) Quantitative analysis of ALP activity; (b) Quantification of ARS

staining. (C-D) ALP staining showing the effects of Y-Exos and si-YOD1 on BMSC osteogenesis, n = 3. (E-F) Immunofluorescence staining evaluating the effects of Y-Exos and si-YOD1 on BMM polarization, n = 4. (G-J) Western blot analysis of osteogenic and macrophage polarization–related protein expression in BMSCs and BMMs treated with Y-Exos and si-YOD1, n = 3. All data are presented as mean ± standard deviation (SD). *P < 0.05; NS, no significant difference.
